# The Transcriptional Repressor Kaiso Localizes at the Mitotic Spindle and Is a Constituent of the Pericentriolar Material

**DOI:** 10.1371/journal.pone.0009203

**Published:** 2010-02-15

**Authors:** Adelheid Soubry, Katrien Staes, Eef Parthoens, Sam Noppen, Christophe Stove, Pieter Bogaert, Jolanda van Hengel, Frans van Roy

**Affiliations:** 1 Department for Molecular Biomedical Research, VIB, Ghent, Belgium; 2 Department of Biomedical Molecular Biology, Ghent University, Ghent, Belgium; Universidade Federal do Rio de Janeiro (UFRJ), Brazil

## Abstract

Kaiso is a BTB/POZ zinc finger protein known as a transcriptional repressor. It was originally identified through its *in vitro* association with the Armadillo protein p120ctn. Subcellular localization of Kaiso in cell lines and in normal and cancerous human tissues revealed that its expression is not restricted to the nucleus. In the present study we monitored Kaiso's subcellular localization during the cell cycle and found the following: (1) during interphase, Kaiso is located not only in the nucleus, but also on microtubular structures, including the centrosome; (2) at metaphase, it is present at the centrosomes and on the spindle microtubules; (3) during telophase, it accumulates at the midbody. We found that Kaiso is a genuine PCM component that belongs to a pericentrin molecular complex. We analyzed the functions of different domains of Kaiso by visualizing the subcellular distribution of GFP-tagged Kaiso fragments throughout the cell cycle. Our results indicate that two domains are responsible for targeting Kaiso to the centrosomes and microtubules. The first domain, designated SA1 for spindle-associated domain 1, is located in the center of the Kaiso protein and localizes at the spindle microtubules and centrosomes; the second domain, SA2, is an evolutionarily conserved domain situated just before the zinc finger domain and might be responsible for localizing Kaiso towards the centrosomal region. Constructs containing both SA domains and Kaiso's aminoterminal BTB/POZ domain triggered the formation of abnormal centrosomes. We also observed that overexpression of longer or full-length Kaiso constructs led to mitotic cell arrest and frequent cell death. Knockdown of Kaiso accelerated cell proliferation. Our data reveal a new target for Kaiso at the centrosomes and spindle microtubules during mitosis. They also strongly imply that Kaiso's function as a transcriptional regulator might be linked to the control of the cell cycle and to cell proliferation in cancer.

## Introduction

Kaiso was discovered a decade ago through its *in vitro* association with the Armadillo protein p120 catenin (p120ctn) [1], a cytoplasmic protein that contributes, via E-cadherin stabilization, to maintenance of cell-cell adhesion in epithelial cells [2], [3]. p120ctn presumably acts both as a tumor suppressor and as a metastasis promoter [3], [4].

Kaiso is a member of the BTB/POZ (Broad Complex, Tramtrak, Bric à brac/Pox virus and Zinc finger) protein family [1], generally known as a group of transcriptional repressors. These proteins contain an N-terminal POZ domain responsible for dimerization and corepressor interactions, and a C-terminal zinc finger domain responsible for DNA association. Kaiso was described as a nuclear protein and appears to be unique in possessing a dual DNA-binding mechanism, as it recognizes methylated CpG dinucleotides as well as a sequence-specific consensus site, CTGCNA, called the Kaiso-binding site or KBS [5]-[7]. Direct gene targets, mostly associated with cancer and/or embryonic development, have been identified for both of these types of DNA interactions. Examples of possible targets for repression by Kaiso are *CDKN2A* [8], *MMP7* [9], *Rb* [10], *MTA2* [5], *E-cadherin, mts-1* and *Xist* [6] in mammals, and *Cyclin D1, Siamois, c-Fos, c-Myc* [11] and *Wnt-11* [12] in *Xenopus.* Notably, Kaiso was also described as a transcriptional activator of the human and mouse *Rapsyn* promoter [13].

The role of Kaiso in embryogenesis is still unclear. Kim et al. [12] reported cross-species conservation of the Kaiso-binding site and the importance of an intact KBS for Kaiso-mediated repression of Wnt11. But Ruzov et al. [14] questioned the role of this putative consensus site in Kaiso's regulation of canonical and non-canonical Wnt gene targets during gastrulation. Instead, they suggested a global repression of methylated genes by Kaiso [14], [15]. Further, Kaiso seems to be dispensable in mammalian embryogenesis [16], [17].

Kaiso's importance in cancer development has also not been fully elucidated. Kaiso's discovery as a nuclear DNA-associating protein and as a binding partner of the well described membrane protein p120ctn inspired researchers to speculate that a Kaiso-p120ctn complex might play an important role in cancer [18]. Reportedly, p120ctn can be present in the nucleus of E-cadherin depleted cells [19], and the association of Kaiso with p120ctn seems to relieve Kaiso's repression of cancer related genes *in vitro* [9], [20], [21]. In gastric epithelial cells, aberrant localization of p120ctn in the nucleus in response to *Helicobacter pylori* infection counteracts Kaiso's repression of the tumor initiating protein matrix metalloproteinase (MMP7) [9]. Earlier findings suggested that Kaiso mediates transcriptional repression of *mmp-7* (*Matrylisin*) in colonic epithelial cells [21]. In general, like other POZ-ZF proteins, Kaiso dysfunction has been linked directly or indirectly to tumor development [22]. Although these reports suggest that Kaiso might protect against cancer by repressing oncogenes, several other findings suggest that Kaiso might also promote tumor formation by repressing tumor suppressor genes. Onset of intestinal cancer in Kaiso-deficient mice is delayed compared to wild-type mice [16]. Human colon tumors show a relatively strong Kaiso expression [16] compared to the normal colon epithelia, which are Kaiso-negative in the nucleus [16], [23]. Kaiso contributes to silencing of tumor suppressor genes, such as *CDKN2A* in colon cancer, and in this way grants cancer cells a survival advantage [8].

Most researchers try to elucidate a role for Kaiso in the nucleus. However, this protein is often not detected in the nuclei of different human tissues, dense cell cultures, and three-dimensional cultures [23]. On the contrary, Kaiso has been observed in the cytoplasm of several normal and cancerous tissues, where it might play a different and yet undiscovered role [23]–[25]. When we previously compared primary ovary tumors to their liver metastases, we observed a change from a weak to a more prominent cytoplasmic expression [23]. A comparable increase in cytoplasmic Kaiso expression has been found in metastases of lung adenocarcinomas and lung squamous cell carcinomas, and was associated with poor prognosis [24], [25]. We described weak to strong Kaiso expression in the cytoplasm of cancer cells, whereas nuclear Kaiso was often not detectable in a variety of human tumors [23]. Strong nuclear Kaiso expression is seen only occasionally in tumors, mostly at tumor-host borders, or in infiltrating parts. Interestingly, changes in the tumor microenvironment lead to translocations of Kaiso within the cell [23]. From these data we hypothesized earlier that there might be a link between Kaiso's transcriptional regulation of cancer-related genes and control of dynamic cell behavior, such as cell migration and loss of differentiation [23].

In this study we strengthened the idea of Kaiso's possible importance in cell dynamics, particularly during cell division. Using different specific antibodies and also different experimental conditions, we showed that Kaiso localizes to cytoplasmic microtubules and centrosomes during interphase and concentrates at the centrosomes and spindle microtubules during mitosis. The spindle is essential for normal cell division in eukaryotic organisms and is composed of a highly dynamic array of microtubules nucleating from two centrosomes and extending to the chromosomes [26]. During the past decade numerous proteins were found to be associated with spindle assembly and significant progress has been made in understanding the dynamic properties of microtubule-associated proteins (MAPs; reviewed in [27]). Nucleating microtubules need to be positioned within the network of centrosomes, which are composed of a pair of centrioles and surrounding pericentriolar material (PCM). Although this process has not been fully elucidated yet, an increasing number of proteins have been reported to be involved. These proteins include γ-tubulin [28], pericentrin [29], NuMA [30], [31], and TPX2 [32].

We demonstrate here the rather unexpected presence of Kaiso at microtubules and centrosomes, mediated by two conserved domains within the protein. We show an association of Kaiso with the PCM constituent pericentrin. Overexpression of some GFP-Kaiso fragments caused abnormal centrosomes and even cell death. Knockdown of Kaiso accelerated cell proliferation. Our present findings reveal that Kaiso is a new component of the centrosomes and the mitotic spindle, where it might play a role in centrosomal duplication or segregation, cell cycle control, and/or microtubule stabilization.

## Materials and Methods

### Cell Culture

Cell lines of human origin were HT29, SW48, SK-LMS-1, MCF-7, MCF-10A, HEK293, HeLa, and MDA-MB-435. Cell lines originating from other species were Ptk-2, L929, HL-1 and MDCK cells. MCF-7 cells were obtained from Prof. Dr. M. Mareel (Ghent University Hospital, Belgium). Other cell lines were purchased from the American Cell Type Culture collection (ATCC, Rockville, MD), with the exception of MCF-10A and HL-1. MCF-10A cells were provided by Dr. J. Brugge (Harvard Medical School, Boston, MA) and grown in a specially supplemented medium as described [33]. HL-1 cells were obtained from Dr. W. Claycomb (Louisiana State University Medical Center, Louisiana, U.S.A.) and grown on fibronectin-coated substrate in appropriate Claycomb's medium supplemented with 10% fetal calf serum (FCS), 10 mM norepinephrine, 0.2 U/ml penicillin, 0.2 mg/ml streptomycin and 2 mM L-glutamate. SK-LMS-1 and Ptk-2 cells were maintained in MEM (Invitrogen, Carlsbad, California) with 10% FCS, non-essential amino acids (Invitrogen), 0.4 mM sodium pyruvate, 0.2 U/ml penicillin, 0.2 mg/ml streptomycin and 2 mM L-glutamate. HT29, HeLa, HEK293, MDA-MB-435, L929 and MCF-7 cells were maintained in DMEM (Invitrogen) with 10% FCS (15% for HT29 and 5% for MCF-7), non-essential amino acids (NEAA; Invitrogen), 0.4 mM sodium-pyruvate, 0.2 U/ml penicillin, 0.2 mg/ml streptomycin, and 2 mM L-glutamate. For MCF-7, the medium was supplemented with 0.01 mg/ml bovine insulin. MDCK cells were grown in a similar medium but without NEAA. SW48 human colon adenocarcinoma cells were maintained in LB15 (Invitrogen) supplemented with 10% FCS, 0.2 U/ml penicillin, 0.2 mg/ml streptomycin and 2 mM L-glutamate.

### Antibodies

Kaiso was detected by using monoclonal antibodies 6F, 12H, 2G and 12G, as well as a rabbit polyclonal antibody (pAb R) raised against a purified 6x-histidine-tagged Kaiso fragment consisting of amino acids (AA) 1–499 (all are gifts from A. Reynolds, Vanderbilt University, Nashville, TN) [34]. Monoclonal antibody 6F was also obtained commercially (Zymed Laboratories Inc., San Francisco). In addition, we used a polyclonal antibody (S1337) that we generated against a region located at the carboxy-terminal end of human and mouse Kaiso (immunogenic peptide: NVTDGSTEFEFIIPESY, AA 655–671) [23]. The specificity of the latter antibody was tested by Western blotting. Purified pre-immune serum and antibody-specific peptide inhibition were used as negative controls. p120ctn was detected by using mAb pp120 (Transduction Laboratories, Lexington, KY) or FITC-conjugated pp120 mAb (Transduction Laboratories), as described [23]. Alpha-tubulin was stained with a rat monoclonal antibody (YOL1/34) diluted 1∶1000 (Abcam, Cambridge, UK). A mouse monoclonal antibody against cyclin B (Transduction Laboratories) was used at a dilution of 1∶250. Centrosomes were visualized by using a gamma-tubulin polyclonal antibody, diluted 1∶200 (Sigma-Aldrich, Saint Louis, Missouri, USA) and a gamma-tubulin monoclonal antibody, diluted 1∶10,000 (Sigma). The PCM was visualized by using the pericentrin-specific rabbit antibody ab4448 (Abcam), diluted 1∶4000.

### Construction of Expression Vectors

Different GFP-tagged Kaiso fragments were constructed by the Gateway cloning technology (Invitrogen). Starting from the eukaryotic expression plasmid pcDNA3-mKaiso (donated by Dr. J. Daniel, McMaster University, Hamilton, Canada), we performed PCRs with primers containing attB1 and attB2 sequences in order to introduce the Kaiso fragments in the entry vector pDONR207 (Invitrogen). We used the following mouse Kaiso fragments cloned in a derivative of pEGFP-C2 (Clontech-Takara, Mountain View, CA, USA) we adapted for Gateway cloning: K1 (aa 1–158), K2 (aa 12–175), K3 (aa 35–501), K4 (aa 64–317), K4x (aa 64–207), K4y (aa 64–225), K4z (aa 195–317), K4a (aa 213–264), K4b (aa 213–287), K4v (aa 213–317), K5 (aa 1–671), K6 (aa 86–490), K7 (aa 1–501), K8 (aa 115–509), and K9 (aa 343–501).

### Synchronization and Transfections

HeLa and HEK293 cells were synchronized by a double thymidine block [35], [36]. They were transiently transfected with Fugene according to the manufacturer's instructions (Roche Diagnostics, Basel, Switzerland). After 12 h they were blocked in early S-phase by incubation for 16 h with 2 mM thymidine (Sigma-Aldrich, St Louis, MO), released for 10 h, and then blocked again for 16 h. The cells entered mitosis 11 h later, and were either fixed or followed by time-lapse microscopy.

### Immunofluorescence

Cells were rinsed briefly with PBS and fixed either with 4% paraformaldehyde in PBS for 25 min at room temperature, or with methanol for 15 min at −20°C (HeLa-GFP-centrin; SK-LMS-1). Immunostaining was performed as described [23]. Cells were incubated with antibodies at 4°C for 1 h or overnight. Secondary antibodies were Alexa488/568/594-coupled anti-mouse or anti-rabbit Ig (Molecular Probes, Eugene, OR). Counterstaining was done with Vectashield mounting medium containing DAPI (Vectorlabs, Burlingame, CA). A Zeiss Axiophot photomicroscope was used for detection, and images were recorded with a MicroMax camera (Princeton Instruments, Trenton, NJ) and Metamorph software (Image Universal Corporation, New York, NY). Time-lapse movies were recorded as described below. Confocal images were made with an inverted Leica SP5 DMI6000 confocal laser scanning microscope.

### Live Cell Microscopy

Cells used in time-lapse microscopy were grown on an eight-well Lab Tek II chamber glass slide (Nunc, VWR International, Leuven, Belgium). Time-lapse microscopy of synchronized transfected cells was carried out using the Leica AS MDW live cell imaging system (Leica Microsystems). This system includes a DM IRE2 microscope equipped with an HCX PL APO 63x/1.3 glycerin-corrected 37°C objective and a 12-bit Coolsnap HQ Camera. The microscope is surrounded by an incubation chamber maintained at 37°C. One hour before imaging, the culture medium was replaced with L15 phosphate buffered medium (Invitrogen), and the cells were placed on the microscope stage to prevent focus drift due to differences in temperature between the Lab Tek chamber and the objective.

DIC and fluorescence images were obtained every 3 min for 5.5 hours. The GFP fluorescence images were monitored by using a monochromator system with a 75-W Xenon lamp for excitation, in combination with a standard B/G/R filter cube. Images were processed into movies using the Leica AS MDW software.

### HeLa-GFP-Centrin Cell Line

HeLa cells were stably transfected by using Amaxa Nucleofector Technology (Lonza Cologne AG, Germany). 1×10^6^ cells were transfected with 2 µg pEGFP-HsCentrin-1 plasmid (kind gift of Dr Bornens, Curie Institute, Paris) [37], using the I-13 program in Nucleofector solution R. Stable transfectants were selected in 600 µg G418/ml (Invitrogen) for 2 weeks and subcloned by limiting dilution.

### Knockdown of Kaiso

For stable knockdown of Kaiso by shRNA, target sequences were cloned in the vector pLV-TH [38]. Briefly, a PCR fragment consisting of part of the H1 promoter, the target sequence (sense), and loop and target sequence (antisense) was generated by two subsequent PCR reactions. A first PCR product, with pSuper as a template, was generated using the forward primer 5′-CTGCAG*GAATTC*GAACGCTGACGTCATCAA-3′ (with an *Eco*RI site indicated in italics) and as reverse primer 5′-AAA*TCTCTTGAA* **TTTAGTAAGACTCTGGTAT** GGGGATCTGTGGTCTCATACAGAACTTATAA-3′. The Kaiso target sequence is indicated in bold; part of the H1 promoter is underlined; the loop sequence is indicated in italics. A thousand-fold dilution of this PCR product was used as a template for a second PCR reaction, with the same forward primer and as a reverse primer 5′-CC*ATCGAT*TTCCAAAAA**ATACCAGAGTCTTACTAAA**TCTCTTGAATTTA-3′ (the *Cla*I site indicated in italics and the Kaiso target sequence in bold). The PCR program consisted of initial denaturation at 94°C for 3 min, followed by 20 cycles of denaturation at 94°C for 1 min, annealing at 55°C for 55 sec, elongation at 72°C for 55 sec, and a final elongation at 72°C for 7 min. This product was subjected to agarose gel electrophoresis, followed by gel extraction (Qiagen), *Eco*RI/*Cla*I digestion, purification (Qiagen) and ligation into *Eco*RI/*Cla*I digested, dephosphorylated pLVTH vector (also purified from gel). After transformation of competent DH5α bacteria with the ligation product, ampicillin-resistant colonies were screened by PCR using primers complementary to sequences of the pLVTH vector surrounding the insert. All constructs were verified by sequencing. They encode transcripts comprising Kaiso-specific shRNA and GFP mRNA. Production of lentiviral particles and transduction of SK-LMS-1 cells was essentially as described before [39]. Transduced cells were sorted (based on high or medium EGFP positivity) using an Epics Altra cell sorter (Beckman Coulter) and named collectively SK-LMS-1_shKAISO.

### Real-Time PCR

RNA was isolated using the RNeasy mini kit (Qiagen) and quality (260/280 nm ratio) and quantity were determined using a Nanodrop® ND-1000 system (Witec AG, Switzerland). For analysis, 400 ng of total RNA was subjected to complimentary DNA (cDNA) synthesis using the iScript cDNA synthesis kit (Bio-Rad Laboratories, Hercules CA) in a volume of 20 µl. Quantitative PCR was performed using the LightCycler 480 system (Applied Biosystems) for 10 ng of complementary DNA with SYBR Green Master mix (Applied Biosystems). For expression of Kaiso we used forward primer 5′-AAGCTTTATCGTTTACATCCAT-3′ and reverse primer 5′-ATACCCAATACCATCATCCTT-3′. Kaiso was normalized to three housekeeping genes: hydroxymethyl-bilane synthase (HMBS), hypoxanthine phosphoribosyl-transferase I (HPRT) and tyrosine 3-monooxygenase/tryptophan 5-monooxygenase activation protein, zeta polypeptide (YWHAZ).

### Sulforhodamine B (SRB) Proliferation Assay

Cells were seeded at 5000 cells/well in 96-well plates and grown for 1 to 14 days. Each test was performed in six replicates. Briefly, cells were fixed by incubation with 50% trichloroacetic acid at 4°C for 1 h or overnight followed by 5 washes with distilled water. Cells were stained by the addition of 1% acetic acid containing 0.4% (w/v) SRB (Sigma) solution to the culture medium at room temperature; after 30 min, plates were washed with 1% acetic acid and air-dried. After the addition of 10 mM Tris-HCl, pH 10.5, to dissolve the SRB bound to cellular proteins, absorbance at 595 nm, proportional to the number of cells attached to the culture plate, was measured by spectrophotometry.

### Flow Cytometric Analysis of Cell Proliferation and Cell Cycle Stage

Proliferation rate and cell cycle status were analyzed for SK-LMS-1_shKAISO cells by monitoring equal dilutions of fluorescently labeled membrane lipids in dividing cells and nuclear Hoechst33342 staining, using a triple-laser (405nm, 488nm, 633nm) LSR-II flow cytometer and FACSDiva software for analysis (Becton Dickinson, NJ). 2×10^6^ cells were labeled using the PKH26 Red Fluorescent Cell Linker Kit (Sigma-Aldrich, MO) according to the manufacturer's instructions. Duplicates of labeled cells were plated in six-well plates at a dilution of 1/2.5 and harvested 24 and 48 h later for analysis. After detachment, washing and counting, cells were stained with 20 µg Hoechst33342 (Sigma-Aldrich)/ml for 60 min at 37°C, and with 2 nM SytoxRed (Molecular Probes, Invitrogen) for 10 min at 37°C, after which the cells were analyzed. Dead cells were excluded on the basis of SytoxRed positivity. Transduced cells with strong shKAISO expression were distinguished from less efficiently transduced cells on the basis of GFP expression. Cell proliferation in GFP+ and GFP–cells was determined by calculating the mean fluorescence intensity (MFI) ratio for PKH26 at 24 h and 48 h.

### Co-Immunoprecipitation (CoIP)

For co-immunoprecipitation of endogenous proteins, cell lysates were prepared in a CoIP solubilization buffer containing 145 mM NaCl, 5 mM EDTA, 2 mM EGTA, 10 mM Tris-HCl pH 7.4, 1% Triton X-100, and a protease inhibitor cocktail (Roche). Lysates were centrifuged and a sample containing 1.25 mg protein was precleared with 25 µl Dynabeads protein G (Invitrogen), and incubated for 4 h or overnight with 3 µg of antibody S1337. 50 µl Dynabeads protein G were then added for 1 h, after which the beads were washed five times with the DynaMag-2 and CoIP solubilization buffer. Washed beads were combined with sample loading buffer and boiled for 5 min. Samples were analyzed by SDS-PAGE and western blotting. Proteins were visualized using the ECL detection system (Amersham).

## Results

### Kaiso Localizes to Microtubules and Centrosomes

**Figure 1:**
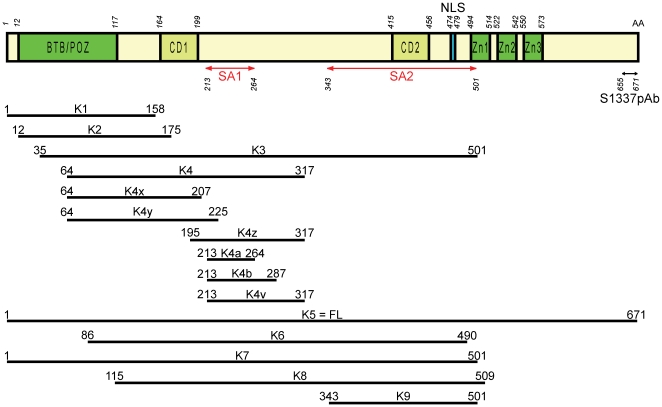
Schematic representation of GFP-tagged Kaiso constructs. Numbers indicate amino acid residues (AA). SA1 and SA2 represent spindle-associated domains. CD1 and CD2 are well-conserved domains (see Suppl. Fig. S1). BTB/POZ, Broad Complex, Tramtrak, Bric à brac/Pox virus and Zinc finger; FL, full-length Kaiso; NLS, nuclear localization signal; pAb, epitope of the polyclonal antibody indicated; Zn, zinc finger domain.

**Figure 2:**
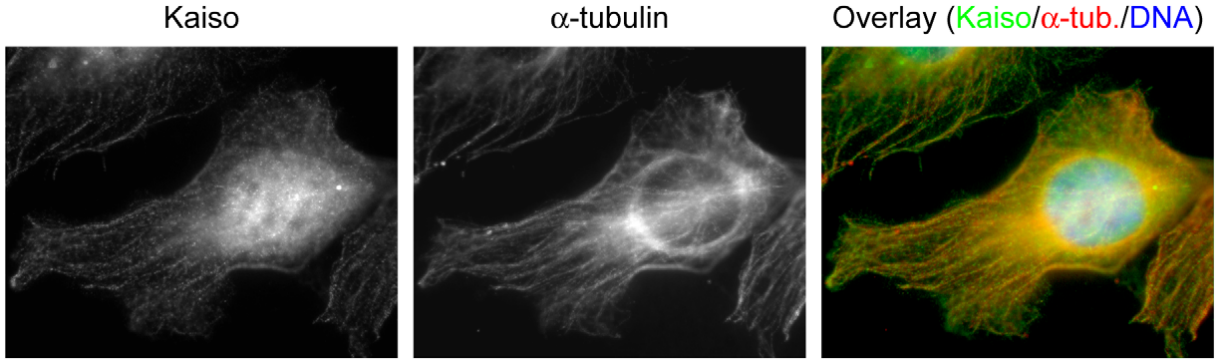
Kaiso colocalizes with microtubules during interphase. Monoclonal antibodies were used to detect the expression of Kaiso (anti-mouse 6F) and alpha-tubulin (anti-rat YOL1/34) in SK-LMS-1 cells. An Alexa 488-conjugated anti-mouse secondary antibody was used in case of Kaiso detection, and an Alexa 594-conjugated anti-rat secondary antibody for alpha-tubulin. DNA was stained with DAPI. Cells were imaged (100× objective lens) with a Zeiss Axiophot microscope while focusing on the cytoplasmic microtubular network.

**Figure 3:**
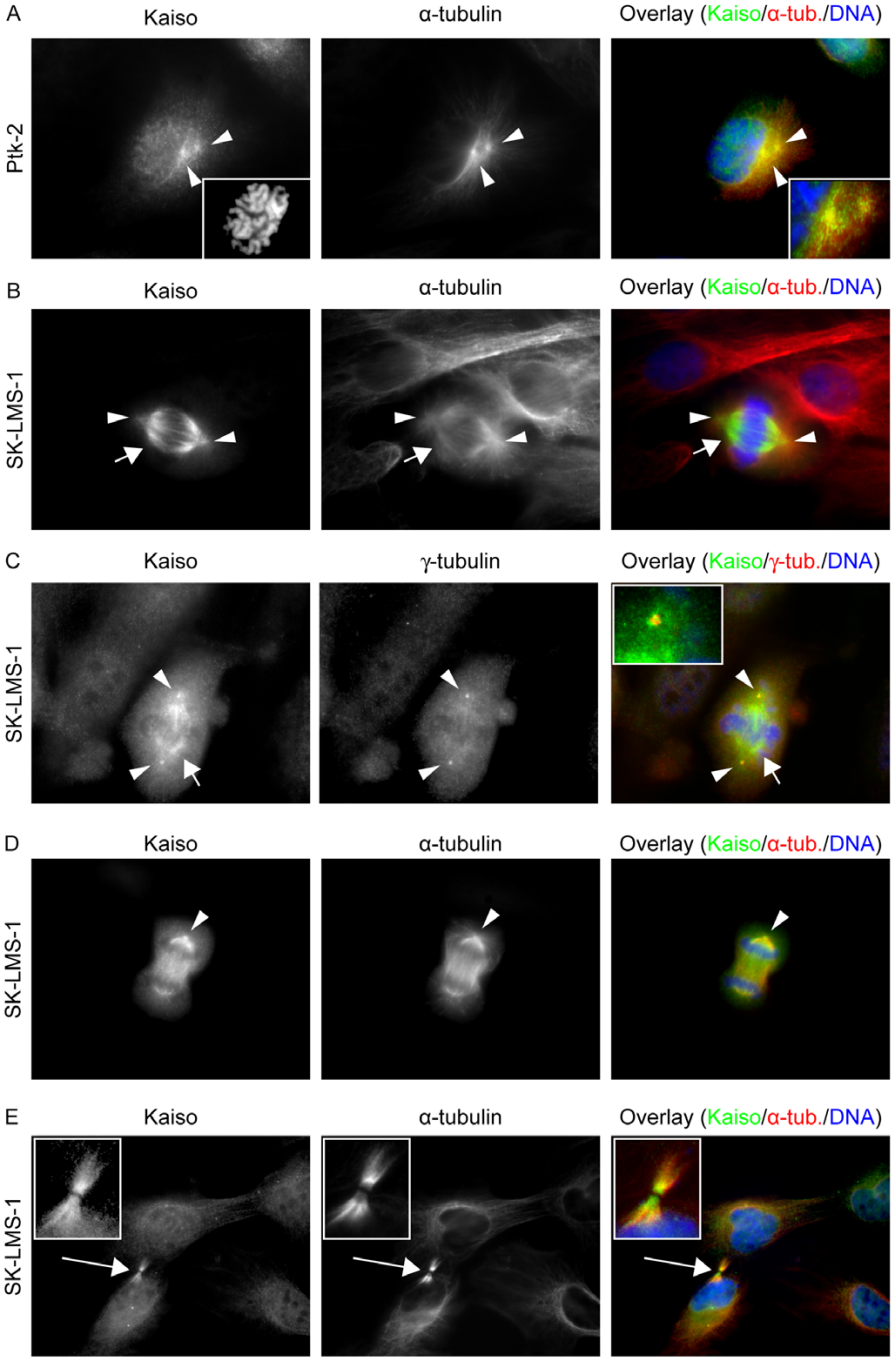
Kaiso localizes at centrosomes and spindle microtubules during mitosis. (A) Staining for Kaiso and α-tubulin in Ptk-2 cells during prophase. Kaiso accumulates at the centrosomal region (arrowheads). The left inset shows chromosomal condensation, while the right inset shows in more detail the colocalization of Kaiso and α-tubulin. During metaphase (B, C) Kaiso is also present on the entire spindle (short arrow). (B) Staining for Kaiso and α-tubulin in SK-LMS-1 cells; photographs were taken while focusing on the spindle microtubules. (C) Localization pattern of Kaiso in SK-LMS-1 cells compared to that of γ-tubulin at the centrosomes; photographs were taken by focusing on centrosomes. The inset magnifies the upper centrosomal region. (D) During anaphase of SK-LMS-1 cells Kaiso localizes at the microtubules and centrosomes, and during cytokinesis (E) it localizes at the midbody (long arrow); insets show magnifications of the midbody region. Monoclonal antibodies were used to detect Kaiso (mouse mAb 6F) or α-tubulin (rat mAb YOL1/34). Secondary antibodies were an Alexa 488-conjugated anti-mouse antibody for detection of Kaiso, and an Alexa 594-conjugated anti-rat antibody for α-tubulin. For γ-tubulin detection, a rabbit polyclonal antibody was used, followed by an Alexa 594-conjugated anti-rabbit secondary antibody. DNA was stained with DAPI. Cells were imaged (100× objective lens) with a Zeiss Axiophot microscope.

The subcellular distribution of Kaiso was followed throughout the cell cycle by immunostaining. Human cell lines SK-LMS-1 and HEK293 were studied with the six Kaiso antibodies described in “Materials and Methods”. In agreement with the literature [1], [34], immunofluorescence microscopy of cells in interphase showed strong Kaiso staining in the nucleus as well as some positive staining in the cytoplasm. All antibodies gave similar results, with the exception of 2G, which also stains cytoplasmic particles previously described as possible artifacts [34]. Because the antibodies 6F, 12H, 2G and pAb R had been raised against the same region (Kaiso AA 1–499) [34], we tried to refine the epitope regions of these antibodies. This was done by designing different GFP-Kaiso expression constructs (Fig. 1). We found that all the available antibodies (commercial and gifts) recognize the same region (AA 213–264), present in fragment K4a, and so we made a new rabbit polyclonal antibody (pAb S1337) specifically recognizing a different Kaiso region, AA 655–671 (see “Materials and Methods”) [23]. In immunofluorescence, pAb S1337 recognized exogenously expressed Kaiso, whereas the staining pattern of endogenous Kaiso in centrosomes was similar to that obtained with the monoclonal antibodies (see below). Specificity was further confirmed by concentration-dependent peptide inhibition (data not shown). We then continued to use mainly antibodies 6F and S1337, which recognize two distinct epitopes in Kaiso. We extended our study to other cells of human origin, such as HT29, SW48, MCF-7, MCF-10A, HeLa and MDA-MB-435 cells, as well as to cells of other species, Ptk-2, L929, HL-1 and MDCK. The different cell lines showed similarly strong Kaiso staining in the nucleus besides variable positive staining in the cytoplasm. The strongest cytoplasmic staining was observed in SK-LMS-1, HeLa and MDCK cells. An image focused on the cytoplasmic content of SK-LMS-1 is shown in Fig. 2; double staining with anti-Kaiso and alpha-tubulin antibodies revealed that the fiber-like staining of cytoplasmic Kaiso colocalized with microtubules. Moreover, Kaiso's distribution was dynamic during cell cycle progression. Fig. 3 shows an overview of the mitotic stages. Kaiso was more concentrated at the centrosomes during prophase (Fig. 3A), and was seen on the entire spindle during metaphase (Fig. 3B–C). Double staining for Kaiso and gamma-tubulin showed partial colocalization at the centrosomes (Fig. 3C). During anaphase Kaiso was distributed uniformly along the elongating spindle and was also present at the centrosomal region (Fig. 3D). At telophase and cytokinesis, Kaiso localized at the midbody, but a region in the division plane was unstained (Fig. 3E). This plane is known to be inaccessible for immunodetection and represents a dense region in which microtubules interdigitate between the two daughter cells [40]. When the intercellular bridge elongated, Kaiso colocalized partly with alpha-tubulin. Kaiso was also seen at the edges (or cell protrusions) of the two forming daughter cells. Finally, Kaiso relocated to the newly formed nuclei and the cytoskeleton. In general, Kaiso staining was more intense at the spindle microtubules during mitosis than at the cytoplasmic microtubules during interphase. In Fig. 3 only Ptk-2 and SK-LMS-1 cells are presented, but all cell lines analyzed showed similar patterns, and at least one monoclonal antibody (mostly 6F) and one polyclonal antibody (S1337) were used in each cell line to confirm these results.

### Kaiso Is a Constituent of the PCM

**Figure 4:**
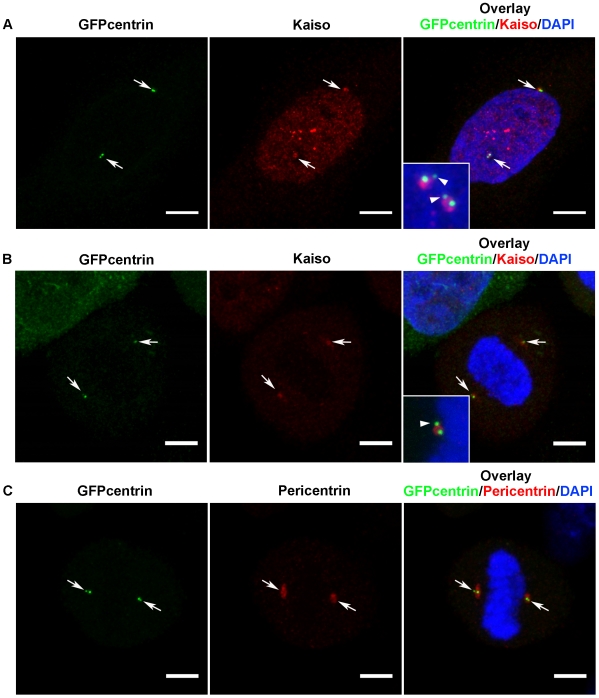
Kaiso localizes in the PCM. Fluorescence microscopy of methanol-fixed HeLa-GFP-centrin cells in either G2/M phase (A) or metaphase (B, C). In (A) and (B) Kaiso was immunolabeled with pAb S1337; in (C) pericentrin was detected by pAb ab4448. Alexa 594-conjugated anti-rabbit secondary antibody was used in all cases and DNA was stained with DAPI. Cells were imaged with a Leica SP5 confocal microscope. Each figure is a projection of a confocal image stack. Colocalization of centrin with Kaiso and pericentrin was confirmed in layers of 0.77 mm thickness. Scale bar, 5 µm. Arrows point at centrosomes. Insets in (A) and (B) show magnified overlay pictures of centriolar regions, each time in a second cell of the same slides. The region of Kaiso-positive staining is broader than the centrin-positive centrioles and overlaps with the mother centriole rather than the daughter centriole (arrowheads). Spindle-associated Kaiso was not obvious in the present experiments, as other fixation and staining conditions were used.

**Figure 5:**
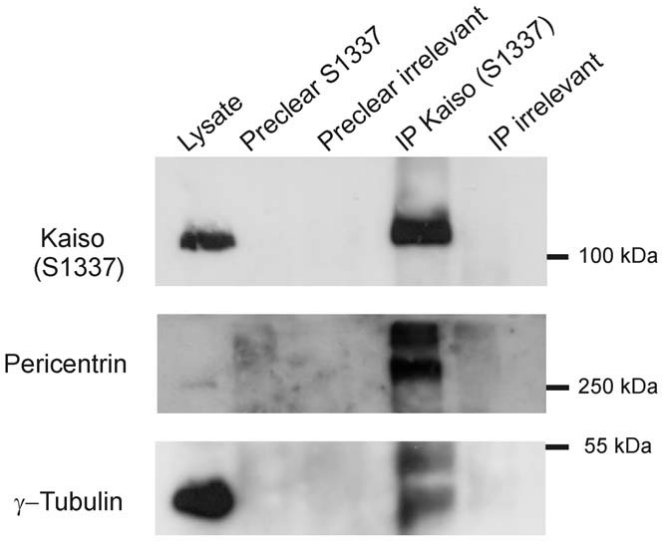
Kaiso associates with PCM proteins in a molecular complex. Lysates were prepared from HeLa-GFP-centrin cells. Immunoprecipitates (IP), using either anti-Kaiso (pAb S1337) or irrelevant antibodies, were separated by SDS-PAGE, followed by western blotting using anti-Kaiso (pAb S1337), anti-pericentrin (pAb ab4448) and anti-γ-tubulin antibodies. Lysate samples are loaded in the left lanes. No specific protein bands were observed in the IP with irrelevant antibody or in the preclear bead eluates. Results are representative of four experiments. It is unclear why the pericentrin and γ-tubulin bands present as doublets. The pericentrin gene is known to encode numerous alternative splice forms [68].

Evidence for specific association of Kaiso with the centrosomes was strengthened by the following observations. Confocal fluorescence images were obtained for HeLa cells expressing GFP-centrin during G2/M and metaphase. A Kaiso-specific signal was invariably observed in close proximity of the tiny GFP-centrin spots in the centrosomes (Fig. 4). Centrin is known to concentrate within the centriole distal lumen [37]. In G2/M cells, this represented only a minor fraction of Kaiso (Fig. 4A), whereas during metaphase Kaiso was largely concentrated in the centriolar regions (Fig. 4B). The Kaiso-specific signals presented as much broader spots than GFP-centrin and encompassed the mother centriole rather than the daughter centriole (Fig. 4, insets). This pattern is reminiscent of the pericentriolar material (PCM), where also pericentrin and ©-tubulin are localized (exemplified in Fig. 4C and Fig. 3C). Similar results were obtained for SK-LMS-1 cells. Further evidence for Kaiso's localization in the PCM was obtained from co-immunoprecipitation experiments. Indeed, for both HeLa-GFP-centrin and SK-LMS-1 cells such experiments reproducibly revealed that a Kaiso-containing molecular complex contained pericentrin (illustrated in Fig. 5). Also ©-tubulin was found in this complex with Kaiso, which is in line with the known interaction of pericentrin with ©-tubulin [41]. All together, this demonstrates that Kaiso is a genuine component of the PCM.

### Identification of Centrosome-Binding and Microtubule-Binding Domains of Kaiso

To further confirm our data obtained by the double stainings above and to determine which domains of Kaiso are required for targeting to microtubules and centrosomes, we carried out expression and functional analysis of several GFP-tagged Kaiso fragments (Fig. 1). We examined the intracellular localization of these different fragments by immunostaining in HEK293, MCF-7, and HeLa cells. The empty GFP vector and another construct encoding an unrelated protein were used as controls.

**Figure 6:**
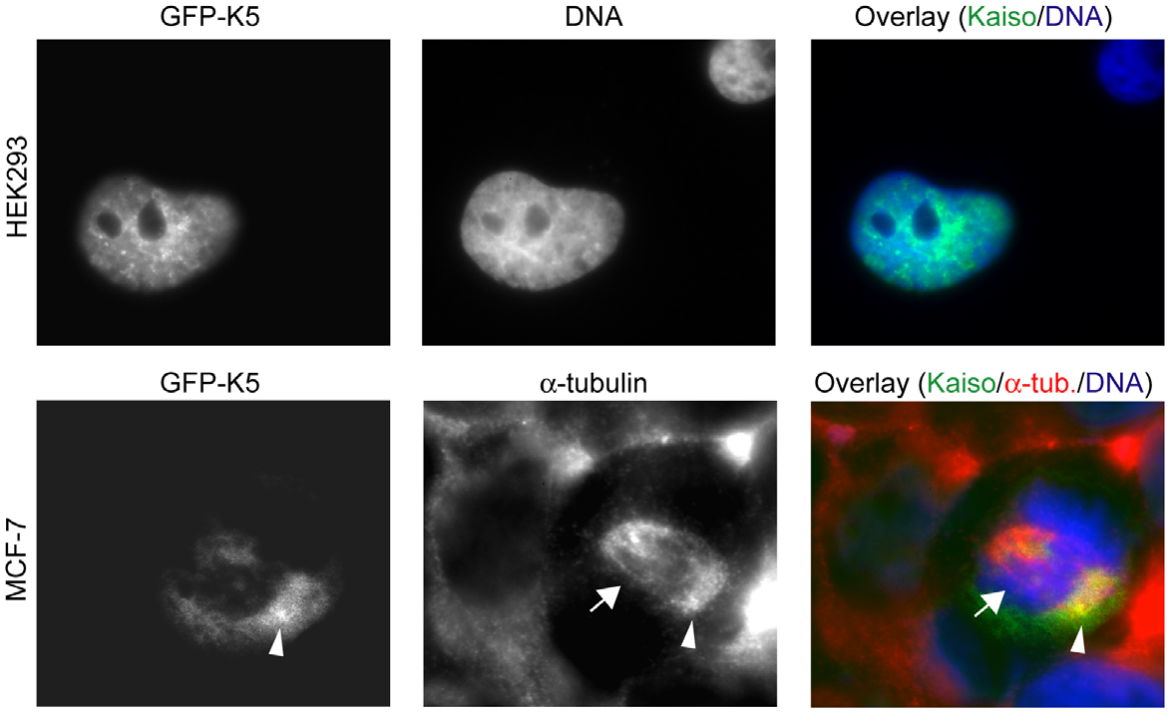
Overexpressed full-length Kaiso localizes in the nucleus during interphase and at spindle material during mitosis. This GFP-tagged full-length Kaiso is encoded by the K5 construct (Fig. 1). It is expressed in the nucleus during interphase (shown for HEK293 cells in upper row) and colocalizes with α-tubulin at the pericentrosomal region during mitosis (shown for MCF-7 in lower row). The picture of an MCF-7 cell in mitosis was made by focusing on the right centrosomal region (arrowhead). The arrow indicates the mitotic spindle. α-tubulin was detected with rat mAb YOL1/34 followed by an Alexa 594-conjugated anti-rat secondary antibody. DNA was stained with DAPI and cells were imaged (100× objective lens) with a Zeiss Axiophot microscope.

During interphase, cells transfected with plasmids encoding GFP-tagged K3, K6, K7, K8 or K9 fragments, or full-length Kaiso (K5), displayed strong Kaiso expression in the nucleus. This is exemplified in the upper row of Fig. 6 for full-length Kaiso in HEK293 cells during interphase. The nuclear expression of the Kaiso fragments is in line with published data about an NLS at position 474–479 [42] (Fig. 1). A second group of Kaiso fragments localized either strongly in the cytoplasm (K4, K4x and K4y), or in both cytoplasm and nucleus (K1, K2, K4a, K4b, K4v and K4z). Remarkably, expression of this group of fragments modified the morphology of the cells, the most prominent feature being the formation of well developed protrusions on single cells (exemplified in Suppl. Fig. S2).

**Figure 7:**
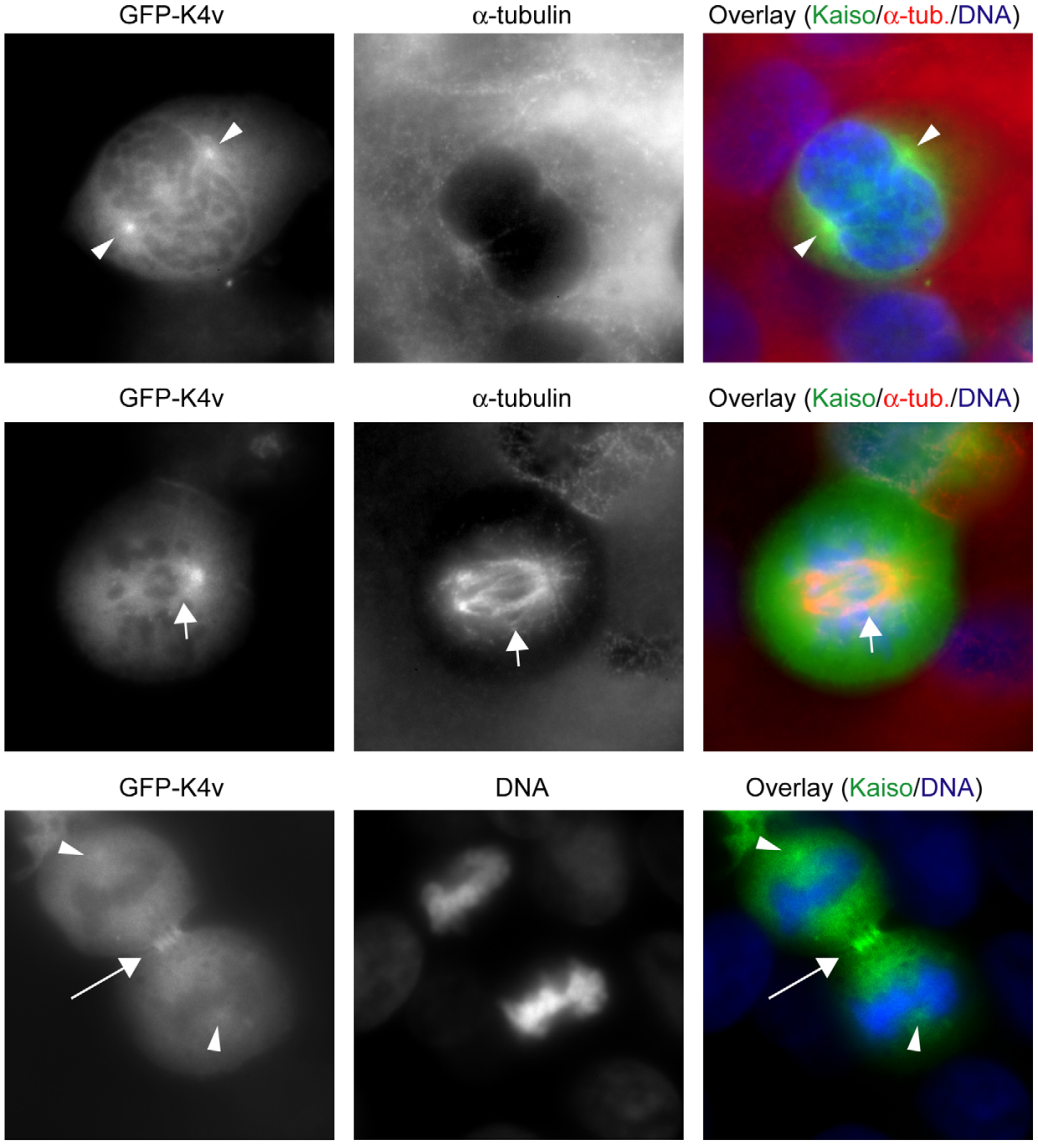
The GFP-tagged Kaiso fragment K4v, comprising SA1, localizes at the spindle material during mitosis. HEK293 cells were transfected with construct GFP-K4v (see also Fig. 1). During the G2/M phase (upper row), GFP-K4v concentrates at the centrosomes (arrowheads). In metaphase (middle row), it is visible along the entire spindle (short arrow). During cytokinesis (lower row), it is present in the midbody (long arrow) and the centrosomes (arrowheads). Rat monoclonal antibody YOL1/34 was used to detect α-tubulin. Secondary antibody was an Alexa 594-conjugated anti-rat antibody. DNA was stained with DAPI. Cells were imaged (100× objective lens) with a Zeiss Axiophot microscope.

**Figure 8:**
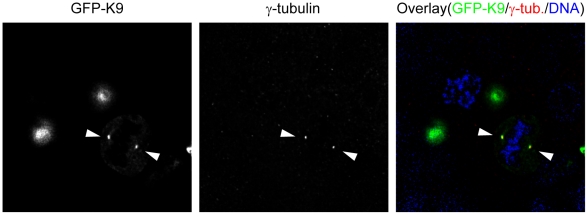
The GFP-tagged Kaiso fragment K9, comprising SA2, localizes at the centrosomes during mitosis. HEK293 cells were transfected with construct GFP-K9 (see also Fig. 1). This fragment localizes only at the centrosomes (arrowheads), which were visualized with a polyclonal anti-γ-tubulin antibody followed by an Alexa 594-conjugated anti-rabbit secondary antibody. DNA was stained with DAPI and cells were imaged (63× objective lens) with a Leica DM IRE2 microscope.

At mitosis, the GFP-tagged protein encoded by the full-length Kaiso construct was distributed at the spindle material. Fig. 6 (lower row) shows colocalization with alpha-tubulin at one of the centrosomes of MCF-7 cells. GFP-tagged Kaiso fragments comprising AA 213–264, such as K4v (Fig. 7), K4z (Suppl. Fig. S3; metaphase and anaphase in upper and lower row, respectively), K4a (Suppl. Fig. S4, upper row) and K4b (Suppl. Fig. S4, lower row), localized at the centrosomes, mainly at the minus ends of the spindle microtubules, whereas fragments without the AA 213–264 domain (such as K1, K2, K4x and K4y) did not localize at the spindle material but disperse throughout the cell (not shown). As an exception, the GFP-tagged fragment K4, containing a conserved region CD1 as well as the 213–264 AA domain (Fig. 1), did not localize at either spindle microtubules or centrosomes. Three substages of mitosis are shown in Fig. 7, which represents HEK293 cells transfected with GFP-K4v. In cells undergoing cytokinesis, we clearly detected GFP-K4v at the midbody (Fig. 7, lower row). In contrast, fragments comprising AA 343–501 (e.g. construct K9) localized mainly at the centrosomes, where they were seen as two intense dots colocalizing with gamma-tubulin (Fig. 8). The figures shown here represent transfections of MCF-7 or HEK293 cells, but other cells studied gave the same results.

Although we do not know which amino acid residues of Kaiso are physically associated with the spindle material, we designated the sequence AA 213–264 as the SA1-domain (for spindle-associated domain 1), and AA 343–501 as the SA2-domain (Fig. 1). In summary, we delineated two functional binding domains of Kaiso involved in localization at spindle microtubules and centrosomes during mitosis.

### Overexpression of Kaiso Leads to Aberrant Centrosomes, Mitotic Arrest and Cell Death

**Figure 9:**
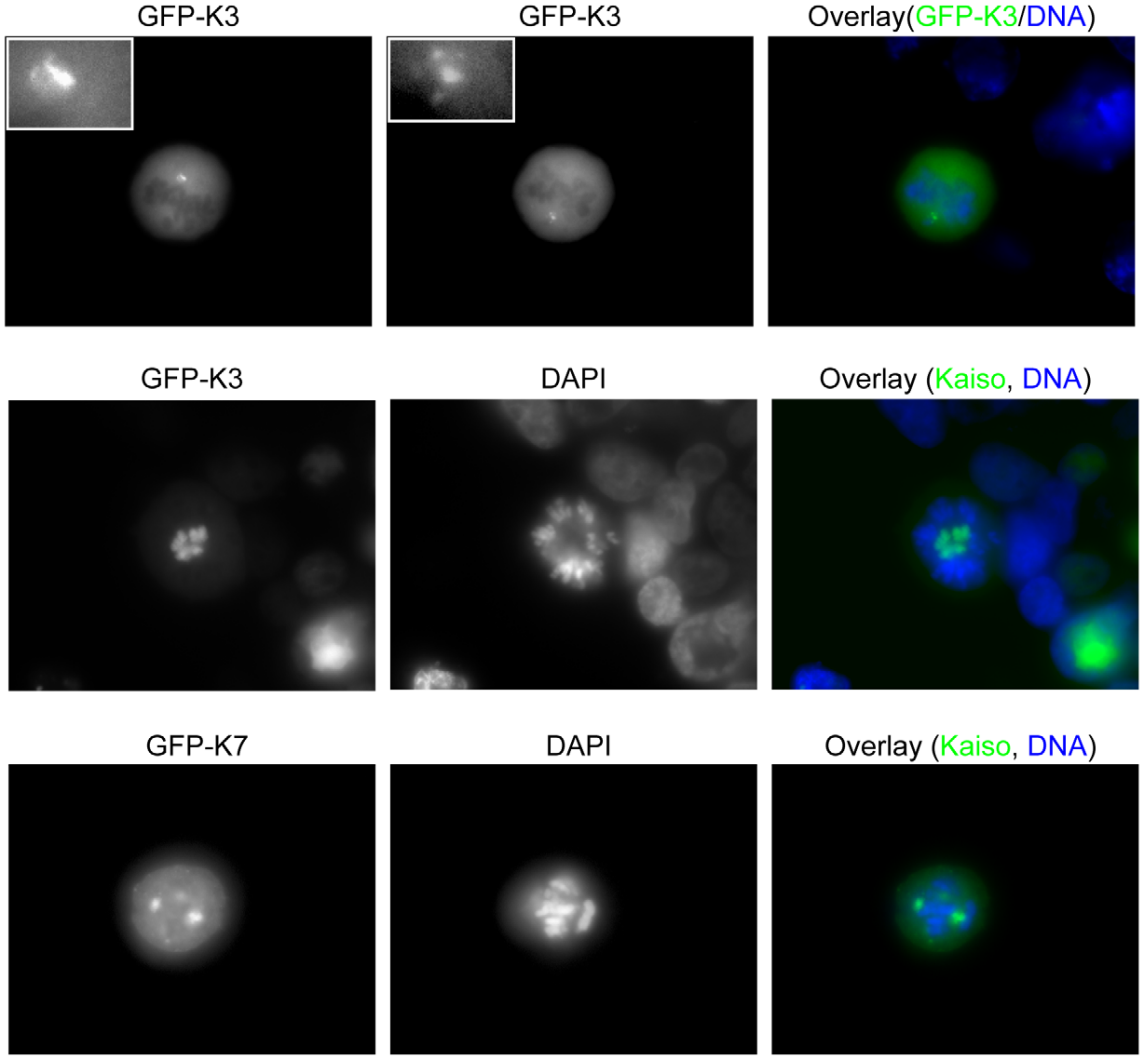
The GFP-tagged Kaiso fragments K3 and K7 abnormally localize at the centrosomes and cause aberrant chromosomal distribution. HEK293 cells were transfected with constructs GFP-K3 and GFP-K7 (see also Fig. 1). In the upper row, cells were imaged by focusing on, respectively, the upper centrosomal region (left) and the lower centrosomal region (middle) (see also insets for magnification of these regions); an overlay of the upper middle picture with DAPI staining to detect DNA is shown on the right. Pictures in the lower two rows also reveal abnormal chromosomal distributions upon overexpression of, respectively, Kaiso fragments K3 and K7. DNA was stained with DAPI. A Zeiss Axiophot microscope was used (100× objective lens).

Time-lapse microscopy was used to follow HEK293 cells transfected with full-length GFP-tagged Kaiso (construct K5) after cell cycle synchronization. Premitotic cell cycle arrest was observed in 95% of the cells. Indeed, mitotic cells were rarely observed (exemplified in Fig. 6), as cells generally started to round up and died before mitosis could start. Time-lapse recording of this process is available online (http://www.dmbr.ugent.be/ext/public/publications/Soubry_2009/GFP_FL-Kaiso.avi) as a Suppl. Movie S1. Sixty hours after transfection, all the cells transfected with the full-length Kaiso were dead. In contrast, transfection with shorter constructs containing the SA1 or SA2 domain but not the complete BTB/POZ or zinc finger domain, such as K4, K8 and K9 (Fig. 1), caused a loss of no more than 10% of the cells. Constructs expressing a complete or nearly complete POZ/BTB, such as K1, K2, K3 and K7 (Fig. 1), caused death of 60 to 95% of the cells. K3 and K7 fragments contain both SA domains, and the surviving fraction of cells overexpressing these fragments showed during mitosis multiple, strong and aberrant dots at putative centrosomal regions (Fig. 9). Aberrant distribution of the chromosomes (Fig. 9, DAPI stain) was accompanied with these abnormally shaped structures.

### Localization of p120ctn during Mitosis

**Figure 10:**
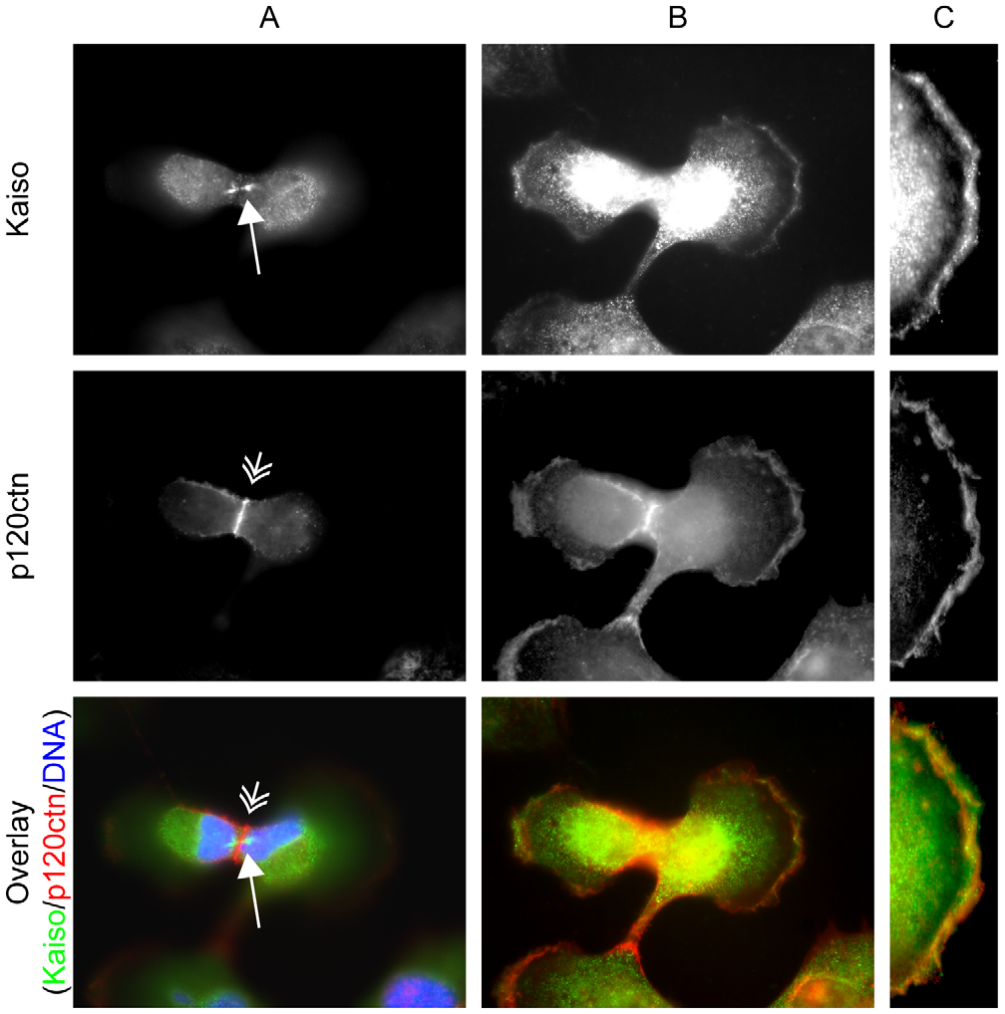
Kaiso and p120ctn localization during cytokinesis. Column A shows pictures made after focusing on the midbody of SK-LMS-1 cells: Kaiso is present at the midbody (long arrow) while p120ctn is located at the cleavage furrow (double short arrow) between the two forming cells. Column B shows pictures of the same cells while focusing on the protrusions at the cell borders. Kaiso and p120ctn partly colocalize there. Pictures in column C are magnifications of the free edge of one of the daughter cells. Cells were stained with a polyclonal antibody (S1337) for Kaiso detection, and with a monoclonal antibody (pp120) for p120ctn detection, followed by an Alexa 488-conjugated anti-rabbit secondary antibody for Kaiso, and an Alexa 594-conjugated anti-mouse secondary antibody for p120ctn. DNA was stained with DAPI. Cells were imaged (63× objective lens) with a Zeiss Axiophot microscope.

The unexpected presence of Kaiso at the mitotic spindle and the centrosomes prompted us to reexamine more precisely the expression of Kaiso's binding partner p120ctn at all stages of mitosis. Using the mouse monoclonal antibody pp120, we did not observe colocalization of endogenous p120ctn with Kaiso at the mitotic spindle or centrosomes in the cell lines HT29, SW48, SK-LMS-1, MCF-7, MCF-10A, HEK293, HeLa, MDA-MB-435, Ptk-2, L929, HL-1 and MDCK. In contrast, we observed endogenous p120ctn during cytokinesis at the cleavage furrow in a ring-shaped form and in the protrusions at the distal edges of the two forming daughter cells (Fig. 10). At this very late stage in cell division, p120ctn and Kaiso largely colocalized at these protrusions but not in the midbody region.

### Knockdown of Kaiso Accelerates Cell Proliferation

**Figure 11:**
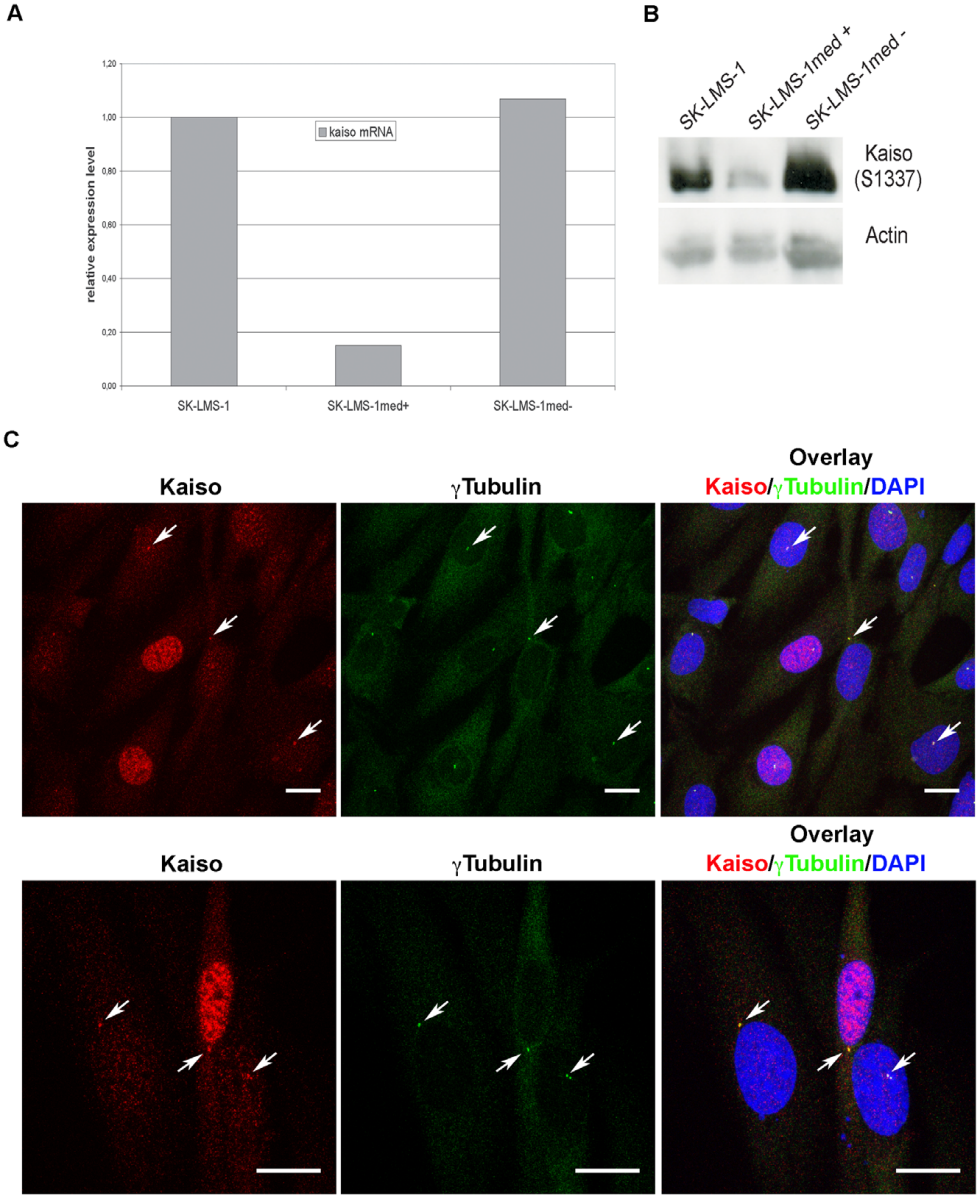
Knockdown of Kaiso in SK-LMS-1_shKAISO cells affects mainly nuclear Kaiso. Parental SK-LMS-1 cells were transduced with lentiviruses encoding transcripts comprising Kaiso-specific shRNA and GFP mRNA. Cells were then FACS sorted and cell populations with medium GFP content were resorted into GFP-positive and GFP-negative cells, named, respectively, SK-LMS-1med+ and SK-LMS-1med–. (A) Relative Kaiso-mRNA expression levels as determined by QRT-PCR. (B) Expression levels of Kaiso protein as determined by western blotting using pAb S1337 and anti-actin for normalization. (C) Immunofluorescent staining for Kaiso and γ-tubulin in SK-LMS-1_shKAISO cell populations in which part of the cells show efficient Kaiso knockdown. In the latter cells diffuse nuclear staining with Kaiso-specific pAb S1337 is gone, whereas staining in centrosomal regions is largely preserved (arrows). Similar results were obtained when staining with mAb 6F. Note that upon methanol treatment the GFP signal disappears from the cells by diffusion. DNA was stained with DAPI and cells were imaged with a Leica SP5 confocal microscope. Each figure is a projection of a confocal image stack. Scale bar, 20 µm.

Next, we attempted to analyze the role of Kaiso in centrosomal functioning during the cell cycle. To this end, we generated SK-LMS-1_shKAISO cells in which a Kaiso-specific shRNA is expressed. The originally transduced cell cultures were FACS sorted for the extent of GFP expression. GFP is coexpressed with the shRNA and is therefore indicative of the stringency of knockdown. Cell populations with medium (med) and high (hi) expression of GFP were resorted, yielding cell populations with robust (labeled +) or weak (labeled –) Kaiso knockdown. For instance, strongly reduced Kaiso mRNA (Fig. 11A) and Kaiso protein (Fig. 11B) were seen in SK-LMS-1med+ cells sorted for medium GFP expression, whereas no Kaiso knockdown occurred in SK-LMS-1med- cells. Double immunofluorescence (Fig. 11C) revealed that the remaining Kaiso protein in cells with efficient Kaiso knockdown preferentially localized in the centrosomal regions. In view of the compelling evidence that Kaiso effectively resides in the PCM, we suggest that the affinity of Kaiso for the PCM is higher than for nuclear components.

**Figure 12:**
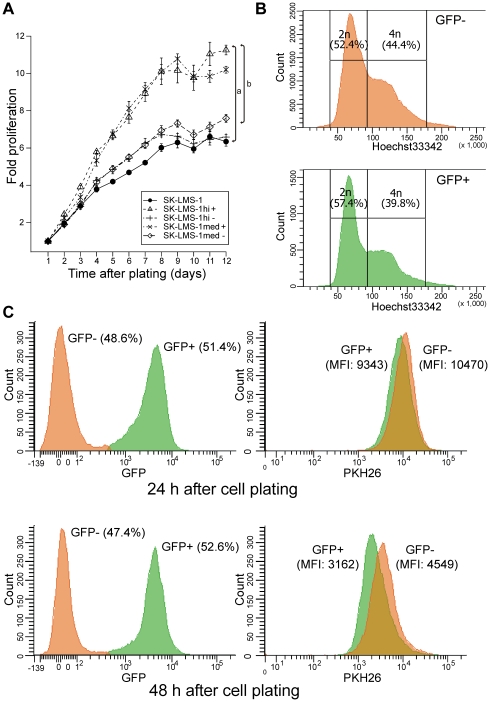
Knockdown of Kaiso in SK-LMS-1 cells accelerates cell proliferation. (A) SK-LMS-1 parental cells and various SK-LMS-1_shKAISO derivative cell populations were subjected to an SRB cell proliferation assay. Cells with medium to high GFP expression levels (med+ and hi+; with stringent Kaiso knockdown) did proliferate more rapidly and reached significantly higher saturation densities than cells with very low GFP expression (hi– and med–; with poor Kaiso knockdown). Values are means ± SD; n = 6. Letters a and b indicate significant differences (p<0.05; Wilcoxon rank sum test): a, SK-LMS-1hi+ vs. parental cells; b, SK-LMS-1hi+ vs. SK-LMS-1hi- cells. (B) Cell cycle profile from unsynchronized SK-LMS-1_shKAISO cell populations with either low GFP expression (GFP–; with poor Kaiso knockdown) or high GFP expression (GFP+, with stringent Kaiso knockdown). Cells were stained with Hoechst33342 and analyzed by flow cytometry to detect 2n/4n DNA contents. In the GFP+ cells, fewer cells (39.8%) contained 4n amounts of DNA than in the GFP– cells (44.4%). Data shown represent one example of four independent experiments. All analyses were performed on subconfluent cell populations. (C) Flow cytometric analysis of cell proliferation by unsynchronized SK-LMS-1_shKAISO cell populations. At time 0, cells were stained with the red fluorescent cell-tracking dye, PKH26, which incorporates into cell membrane lipids and is thus diluted twofold every cell division. At 24 h and at 48 h after plating, cells were first distinguished according to GFP levels (left panels), where cells with high GFP levels (GFP+) show strong Kaiso knockdown. Dead cells were excluded from analysis by positive staining for SytoxRed. Mean PKH26 fluorescence intensity (MFI) was then measured in each subpopulation (right panels), showing that GFP+ cells underwent 1.56 [log_2_(9343/3162)] cell divisions in 24 h, whereas GFP– cells underwent 1.20 [log_2_(10470/4549)] cell divisions.

We then addressed the functional implications of Kaiso knockdown. Abnormalities in mitotic spindle formation, centrosomal division or PCM composition appeared not to be increased by the knockdown (data not shown). However, in a cell proliferation assay, cell populations with efficient Kaiso knockdown showed significantly increased growth rate and saturation density (Fig. 12A). It was somewhat surprising that DNA content analysis in unsynchronized, subconfluent SK-LMS-1_shKAISO cell subpopulations showed a lower level of 4n cells in GFP+ cells with strong Kaiso reduction (Fig. 12B). In combination with the increased growth rate and density of such cells, this points at a shorter S/G2/M period rather than a shorter G1 phase. The length of the cell cycle was analyzed on the basis of dilution speed of PHK26, a lipophilic dye that stably integrates into the cell membrane (Fig. 12C). Subconfluent GFP+ subpopulations of SK-LMS-1_shKAISO cells underwent 1.56 cell divisions in 24 h, whereas 1.20 divisions were calculated for GFP–cells. Hence, in SK-LMS-1 cells Kaiso slows down the cell cycle.

## Discussion

In this study we found a novel localization for the transcription factor Kaiso, suggestive of a new function. We examined the expression patterns of the Kaiso protein and several fragments thereof during the cell cycle and found that Kaiso is present not only in the nucleus, but also at the centrosomes and the mitotic spindle. Localization of Kaiso outside the nucleus of cultivated cells has been referred to only very briefly [1] and the number of reports about its subcellular localization *in vivo* is very limited [23]–[25]. We found that Kaiso's colocalization is dynamic throughout the cell cycle: it localizes at the microtubules during interphase and concentrates at the centrosomes most prominently before and during mitosis. Kaiso distributes along the spindle microtubules at metaphase, and finally localizes at the midbody during cytokinesis. Since the initial submission of our data, a very limited report described Kaiso's presence at centrosomes and midbody [43], which confirms our observations. To demonstrate more thoroughly Kaiso's expression patterns throughout the cell cycle, we used two different methods: antibodies recognizing different epitopes of Kaiso, and transient transfection with different GFP-Kaiso constructs.

**Figure 13:**
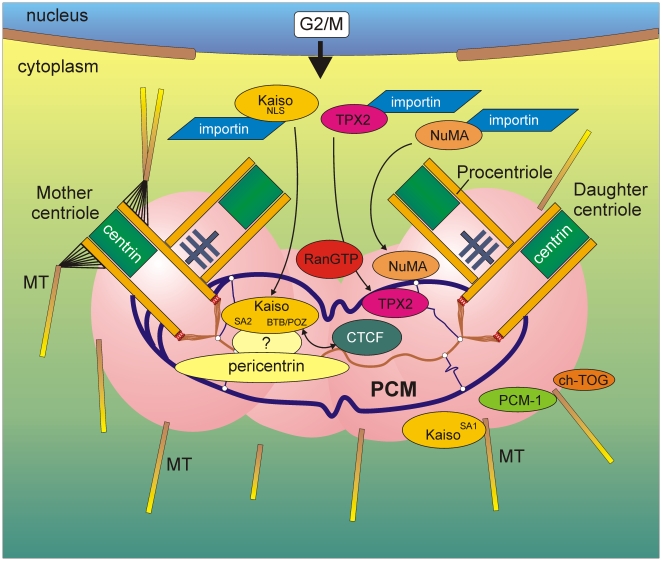
Hypothetical model for interactions of Kaiso's functional domains at the centrosomes during the onset of mitosis. The Kaiso domains putatively responsible for its localization at the centrosomes or spindle microtubules are shown inside yellow Kaiso balloons. At the G2/M phase, the centrosome consists of a mother and a daughter centriole [drawn according to 44], as well as two growing procentrioles. Centrin (depicted in green) is concentrated in the centriolar distal lumen. MT, microtubules.***A role for the SA2 domain of Kaiso:*** After breakdown of the nuclear envelope, nuclear proteins, such as NuMA, TPX2 and possibly also Kaiso, are released in a RanGTP-dependent manner from complexes containing importin. A fraction of RanGTP is present at the centrosomes throughout the cell cycle and can locally activate these centrosomal factors during G2/M phase; this allows microtubule nucleation and stabilization near the centriole pairs [69]. The molecular Kaiso-pericentrin interaction in the PCM, which is direct or indirect via an adaptor (depicted as ?), might also be a function of the SA2 domain.***A role for the SA1 domain:*** Kaiso might associate either directly with the microtubules or indirectly *via* the motor protein dynein, along with other centrosomal components such as PCM-1 and ch-TOG.***A role for the BTB/POZ domain in the centrosome:*** The zinc finger protein CTCF might recruit Kaiso towards the centrosomes (or *vice versa*) *via* the hetero-dimerization capacities of Kaiso's BTB/POZ domain.

Immunofluorescent colocalization revealed that Kaiso is less focally expressed in the centrosomes than centrin. Evidence for association of Kaiso with the PCM was strengthened by our finding of pericentrin and ©-tubulin in a molecular complex with Kaiso. PCM is a structurally intricate protein complex consisting of a lattice of large coiled coil proteins, including pericentrin and AKAP-450 [44], [45] (Fig. 13). Interestingly, pericentrin associates with γ-tubulin to form a complex that controls spindle organization and mitotic entry [29]. Moreover, pericentrin interacts with many other structural and regulatory proteins (reviewed in [41]), including the chromatin remodeling proteins CHD3 and CHD4 [46]. Inactivating mutations of pericentrin lead to cell cycle checkpoint and microtubule organization defects, resulting in mitotic arrest, dwarfism and ciliopathies, whereas pericentrin overexpression is seen in cancers and correlates with chromosomal instability (reviewed in [41]). The occurrence of Kaiso, a transcriptional repressor, as a novel pericentrin interactor suggests an intriguing regulatory role for both pericentrin and Kaiso, but the details of this interaction await further study.

From our transient transfection experiments we identified at least two domains within Kaiso that appear to be responsible for its localization at the mitotic spindle and/or centrosomes, and designated them SA1 and SA2 (for spindle-associated-1 and -2). We also observed that transfection with particular constructs can result in abnormal centrosome shapes and aberrant mitotic cleavage. Kaiso fragments containing the SA1 domain (*e.g*. K4a, K4b, K4v and K4z) concentrated at the centrosomes, spindle microtubuli and midbody of mitotic cells. In contrast, fragments such as K4, containing also CD1, a conserved acidic region just before SA1, did not localize at the centrosomes. We assume that in some protein constructs CD1 causes conformational changes in such a way that SA1 is kept off the centrosomes. SA2-containing fragments showed remarkably strong expression at the centrosomes during mitosis (*e.g*. K9), but not at the spindle microtubules. The differences seen between constructs containing SA1 and SA2 might indicate that they bind differently to the mitotic spindle components: either transported along the microtubules by motor proteins (in case of SA1), or by binding directly to centrosomal components (in case of SA2). A hypothetical model of the binding mechanisms and possible functions of Kaiso in mitosis is presented in Fig. 13.

The SA2 region comprises at least two potentially interesting domains. First, there is the yet uncharacterized domain CD2, conserved from mammals to chicken, frog and zebrafish (Suppl. Fig. S1). This suggests that the AA 412–453 sequence within the SA2 domain is functionally important in positioning Kaiso at the centrosomes during mitosis. Second, the SA2 domain includes a Nuclear Localization Sequence (NLS; AA 474–479; Fig. 1) shown to be required for its import into the nucleus [42]. The main proteins involved in such nuclear translocation are importin-〈2 and Ran-GTP, and a direct interaction between Kaiso and importin-〈2 has indeed been reported, both *in vivo* and *in vitro* [42]. Interestingly, other NLS containing proteins, such as NuMA and TPX2, have also been localized at the centrosomes and mitotic spindle. They are reported to be spindle assembly factors (SAF) and are regulated by Ran and importin [32], [47], [48] (Fig. 13). It is conceivable that Kaiso's functional NLS is at the root of the relocalization of the bulk of nuclear GFP-Kaiso to the centrosomes after breakdown of the nuclear envelope during mitosis. The dissociation of importin from the NLS domain of Kaiso might also make it possible for a nearby centrosome-binding domain (within SA2; Fig. 1) to bind to centrosomal components, which is otherwise hindered by importin. Although this is still hypothetical, similar mechanisms have been elucidated for the motor protein kinesin-14 XCTK2 [49] and for the kinesin-like DNA binding protein Kid [50], [51]. In view of the identification of Kaiso as another SAF, a role needs to be considered for GTP-Ran in the localization and/or activity of Kaiso at the spindle microtubules or centrosomes (Fig. 13).

Other transcription regulating zinc finger proteins have also been related to the centrosomes. The zinc finger protein basonuclin is present in the nuclei of mitotically active epidermal cells, as well as in the centrosomes of meiotic spermatocytes [52]. In *Drosophila*, CP190 and CTC-binding factor (CTCF) are insulator zinc finger proteins and mutual binding partners, associating with chromatin during interphase and with centrosomes in mitotic cells (Fig. 13) [53]–[56]. However, neither the region responsible for the centrosomal localization of these proteins nor the underlying mechanism has been elucidated yet. Interestingly, CTCF was described as a Kaiso binding partner [57]. Their mutual interaction occurs through the C-terminal region of CTCF and the N-terminal BTB/POZ domain of Kaiso. The presence of multiple zinc finger proteins (including Kaiso) at the spindle apparatus might indicate that they cooperate in cell growth and supports the notion of their possible importance in the formation, positioning or function of centrosomes [52].

Another region that might be important for targeting Kaiso to the centrosomes is the BTB/POZ domain. This highly conserved domain (Suppl. Fig. S1) promotes heteromeric dimerization, which might contribute to recruitment of Kaiso towards the centrosomes by centrosomal factors, such as the abovementioned CTCF (Fig. 13) and pericentrin. The possible influence of Kaiso's BTB/POZ domain on important cellular processes is strongly indicated by two of our observations. First, transfection of cells with fusion constructs encompassing the BTB/POZ domain, such as GFP-K3 or GFP-K7, results in aberrant centrosomal shapes and organization, including monopolar spindles (Fig. 9). Second, overexpression of full-length Kaiso containing an intact BTB/POZ domain leads generally to cell death. Unfortunately, we could not obtain enough mitotic cells for in depth molecular analysis of this phenomenon. It is likely, however, that the level of Kaiso must be tightly regulated during the normal cell cycle.

The fact that different domains might be responsible for targeting Kaiso towards the spindle indicates that the functioning of Kaiso is relatively complex. The contribution of each domain can vary slightly with time and in space. But Kaiso is not the only protein with more than one spindle-associating domain; for example, XMAP215 in *Xenopus* has also been reported to use two domains, namely its C-terminal domain to target centrosomes and microtubules, and its evolutionarily conserved N-terminal domain, which contains a microtubule-stabilizing activity [58]. The human homolog of XMAP215, ch-TOG, plays a major role in maintaining the integrity of the spindle poles [59] (Fig. 13). Interestingly, several centrosomal proteins, including PCM-1, ninein, centrin and pericentrin, also localize in so-called pericentrosomal satellites [44], a process that is dynein/dynactin-dependent [60], [61] (Fig. 13). Immunostaining patterns for PCM-1 throughout the cell cycle [61] are reminiscent of those of Kaiso (our unpublished data and ref. [23]). We hypothesize that the SA1 domain is responsible for Kaiso's localization at the microtubules during interphase and mitosis. This association might be either direct or indirect as part of the PCM-1/dynactin or the pericentrin/dynein-containing transport complexes (Fig. 13).

It was reported that the Kaiso's interaction partner p120ctn localizes at the microtubules and centrosomes of MDA-MB-231 breast carcinoma cells, especially after overexpression of a particular p120ctn mutant. From CoIP experiments, Franz and Ridley concluded that the interaction must be indirect [62]. We propose that Kaiso could be the undiscovered link. Chartier et al. (2007) showed that the overexpressed p120ctn isoform 3A accumulates at the centrosomes of mitotic HT29 cells [63]. Interestingly, isoform 3A rather than isoform 1A has been proposed to directly interact with Kaiso [25]. Our centrosome binding Kaiso fragments containing either SA1 or SA2 (K9, K4a, K4b, K4v and K4z) do not include the C-terminal p120ctn-binding domain [1]. Hence, full-length Kaiso may be able to bind simultaneously to centrosomes and to isoform 3A of p120ctn. Moreover, if p120ctn binding to Kaiso interferes with Kaiso's normal function at the centrosomes, it might also explain the hyperduplication of centrosomes in cells overexpressing p120ctn 3A [63]. The disturbance in the amount or the distribution of subcellular p120ctn seen in many cancers [4], [25], [64], [65] may lead to excessive accumulation of p120ctn at the centrosomes and consequent disturbance of the function of Kaiso. We earlier reported a coupled increase of cytoplasmic p120ctn and Kaiso *in vivo* when primary tumors became invasive and formed metastases [23]. Notably, stronger cytoplasmic Kaiso expression, often associated with abnormal cytoplasmic expression of p120ctn, was recently shown to be related to poor prognosis of non-small-cell lung cancer [24], [25]. The previously detected but undefined dot-like Kaiso-positive structures around the nuclei of inflammatory breast cancer cells and in muscle cells of the colon, as well as the strong cytoplasmic staining of ciliated cells, might be an indication of centrosome-related Kaiso expression *in vivo* [23]. We also have indications that Kaiso might be present at the basal bodies of ciliated cells in the bronchus (our unpublished data), where also pericentrin is localized [41].

Aiming to further elucidate the role of Kaiso in cell division, we knocked down Kaiso expression in SK-LMS-1 cells. Knockdown is never complete, and from repeated experiments on cells enriched for high shRNA expression, it turned out that centrosome-associated Kaiso was particularly resistant to this manipulation. On the other hand, this resistance might imply that the affinity of Kaiso for the PCM is higher than for nuclear components. The knockdown achieved resulted in increased proliferation rate, increased saturation density, and shortened cell cycle. This is in line with the observation of cytoplasmic rather than nuclear Kaiso in progressed tumors [23]–[25]. Nuclear Kaiso is known to repress various tumor-associated β-catenin target genes, such as *Ccnd1*, which encodes cyclin D1 (reviewed in [66]). Hence, knockdown of nuclear Kaiso is expected to counteract this repression. Alternatively, we cannot exclude the involvement of slight repression of centrosomal Kaiso in the observed growth stimulation. One might speculate that centrosomal Kaiso also suppresses growth by interacting with the proto-oncogenic pericentrin [41]. Narrowing down to key residues in the SA1 and SA2 regions, as well as the putative pericentrin binding domain, followed by knock-in of site-specific mutations may be a feasible approach to address these questions.

In summary, we describe a detailed analysis of the transcription factor Kaiso during cell cycle progression. We observed Kaiso at the mitotic spindle apparatus, and we delineated at least two domains, SA1 and SA2. The former is responsible for targeting Kaiso to interphase and mitotic microtubules, whereas the latter targets it to the PCM in centrosomes. We also underlined the possible importance of the BTB/POZ domain. We demonstrate that overexpression of certain Kaiso fragments leads to aberrant centrosomal shapes, chromosomal disarrangement, mitotic arrest, and even cell death. Our observations point to a new functional role for Kaiso in centrosome separation during mitosis, microtubule nucleation, and/or the G2-M checkpoint. Our observation of Kaiso in a molecular complex with pericentrin should be scrutinized further. Although Kaiso's role in tumorigenesis remains poorly understood, suggestions have been made to use Kaiso as a therapeutic target in cancer [16], [67]. We believe that our present findings should be taken into careful consideration before such efforts are undertaken.

### Supplementary Data

Supplementary data associated with this article can be found online at http://www.dmbr.ugent.be/ext/public/publications/Soubry_2009/GFP_FL-Kaiso.avi.

## Supporting Information

Figure S1 Alignment of Kaiso proteins of various species reveals two conserved domains, CD1 and CD2, in addition to the BTB/POZ and zinc finger domains. Multiple sequence alignment of Kaiso protein sequences from mammals (human, chimpanzee, rhesus monkey, horse, mouse, rat) and other vertebrates (chicken, the frog Xenopus laevis, zebrafish). RefSeq sequences were obtained from the Entrez Gene database [1] by searching for “Kaiso OR Zbtb33” and aligned with ClustalX2 using default parameters [2]. The shaded background was created with the BoxShade server. The line below every alignment block denotes the type of conservation: “*” for identical residues, “:” for high similarity and “.” for weak similarity. Annotation of the BTB/POZ domain (cyan background) and zinc fingers (green background; red: C and H residues) was based on, respectively, CD-Search [3] and InterPro [4] analyses. Other conserved regions, CD1, CD2 and NLS, are indicated by yellow background. The novel Spindle-Associated domains 1 and 2 (SA1 and SA2) are highlighted in pink. See also Fig. 1 for domain organization. References [1]: Maglott D, Ostell J, Pruitt KD, Tatusova T (2007) Entrez Gene: gene-centered information at NCBI. Nucleic Acids Res 35: D26-31. [2]: Larkin MA, Blackshields G, Brown NP, Chenna R, McGettigan PA, et al. (2007) Clustal W and Clustal X version 2.0. Bioinformatics 23: 2947–2948. [3]: Marchler-Bauer A, Bryant SH (2004) CD-Search: protein domain annotations on the fly. Nucleic Acids Res 32: W327-331. [4]: Hunter S, Apweiler R, Attwood TK, Bairoch A, Bateman A, et al. (2009) InterPro: the integrative protein signature database. Nucleic Acids Res 37: D211-215.
(0.18 MB PDF)

Figure S2 The GFP-tagged Kaiso fragment K4x causes formation of protrusions during interphase. HEK293 cells are transfected with construct GFP-K4x. DNA was stained with DAPI, and cells were imaged with a Zeiss Axiophot microscope (100× objective lens).
(0.18 MB PDF)

Figure S3 The GFP-tagged Kaiso fragment K4z localizes at spindle material during mitosis. During metaphase (upper row) and anaphase (lower row) of transfected HEK293 cells, the Kaiso fragment K4z concentrates at the centrosomes (arrowheads) and the minus ends of the spindle microtubules (arrows). DNA was stained with DAPI and cells were imaged with a Leica DM IRE2 microscope (63× objective lens).
(1.59 MB PDF)

Figure S4 The GFP-tagged Kaiso fragments K4a and K4b localize at the spindle material during mitosis. In transfected HEK293 cells the K4a (upper row) and K4b (lower row) Kaiso fragments both localize at the spindle microtubules (arrows) and at the centrosomes (arrowheads). DNA was stained with DAPI and cells were imaged with a Zeiss Axiophot microscope (100× objective lens).
(0.70 MB PDF)

Movie S1 (3.41 MB AVI)

## References

[1] Daniel JM, Reynolds AB (1999). The catenin p120(ctn) interacts with Kaiso, a novel BTB/POZ domain zinc finger transcription factor.. Mol Cell Biol.

[2] Reynolds AB, Daniel J, McCrea PD, Wheelock MJ, Wu J (1994). Identification of a new catenin: the tyrosine kinase substrate p120cas associates with E-cadherin complexes.. Mol Cell Biol.

[3] Reynolds AB, Roczniak-Ferguson A (2004). Emerging roles for p120-catenin in cell adhesion and cancer.. Oncogene.

[4] van Hengel J, van Roy F (2007). Diverse functions of p120ctn in tumors.. Biochim Biophys Acta.

[5] Yoon HG, Chan DW, Reynolds AB, Qin J, Wong J (2003). N-CoR mediates DNA methylation-dependent repression through a methyl CpG binding protein Kaiso.. Mol Cell.

[6] Prokhortchouk A, Hendrich B, Jorgensen H, Ruzov A, Wilm M (2001). The p120 catenin partner Kaiso is a DNA methylation-dependent transcriptional repressor.. Genes Dev.

[7] Daniel JM, Spring CM, Crawford HC, Reynolds AB, Baig A (2002). The p120(ctn)-binding partner Kaiso is a bi-modal DNA-binding protein that recognizes both a sequence-specific consensus and methylated CpG dinucleotides.. Nucleic Acids Res.

[8] Lopes EC, Valls E, Figueroa ME, Mazur A, Meng FG (2008). Kaiso contributes to DNA methylation-dependent silencing of tumor suppressor genes in colon cancer cell lines.. Cancer Res.

[9] Ogden SR, Wroblewski LE, Weydig C, Romero-Gallo J, O'Brien DP (2008). p120 and Kaiso regulate Helicobacter pylori-induced expression of matrix metalloproteinase-7.. Mol Biol Cell.

[10] De La Rosa-Velazquez IA, Rincon-Arano H, Benitez-Bribiesca L, Recillas-Targa F (2007). Epigenetic regulation of the human retinoblastoma tumor suppressor gene promoter by CTCF.. Cancer Res.

[11] Park JI, Kim SW, Lyons JP, Ji H, Nguyen TT (2005). Kaiso/p120-catenin and TCF/beta-catenin complexes coordinately regulate canonical Wnt gene targets.. Dev Cell.

[12] Kim SW, Park JI, Spring CM, Sater AK, Ji H (2004). Non-canonical Wnt signals are modulated by the Kaiso transcriptional repressor and p120-catenin.. Nat Cell Biol.

[13] Rodova M, Kelly KF, Van Saun M, Daniel JM, Werle MJ (2004). Regulation of the rapsyn promoter by kaiso and delta-catenin.. Mol Cell Biol.

[14] Ruzov A, Hackett JA, Prokhortchouk A, Reddington JP, Madej MJ (2009). The interaction of xKaiso with xTcf3: a revised model for integration of epigenetic and Wnt signalling pathways.. Development.

[15] Ruzov A, Dunican DS, Prokhortchouk A, Pennings S, Stancheva I (2004). Kaiso is a genome-wide repressor of transcription that is essential for amphibian development.. Development.

[16] Prokhortchouk A, Sansom O, Selfridge J, Caballero IM, Salozhin S (2006). Kaiso-deficient mice show resistance to intestinal cancer.. Mol Cell Biol.

[17] Martin Caballero I, Hansen J, Leaford D, Pollard S, Hendrich BD (2009). The methyl-CpG binding proteins Mecp2, Mbd2 and Kaiso are dispensable for mouse embryogenesis, but play a redundant function in neural differentiation.. PLoS ONE.

[18] van Roy FM, McCrea PD (2005). A role for Kaiso-p120ctn complexes in cancer?. Nat Rev Cancer.

[19] van Hengel J, Vanhoenacker P, Staes K, van Roy F (1999). Nuclear localization of the p120(ctn) Armadillo-like catenin is counteracted by a nuclear export signal and by E-cadherin expression.. Proc Natl Acad Sci U S A.

[20] Kelly KF, Spring CM, Otchere AA, Daniel JM (2004). NLS-dependent nuclear localization of p120ctn is necessary to relieve Kaiso-mediated transcriptional repression.. J Cell Sci.

[21] Spring CM, Kelly KF, O'Kelly I, Graham M, Crawford HC (2005). The catenin p120ctn inhibits Kaiso-mediated transcriptional repression of the beta-catenin/TCF target gene matrilysin.. Exp Cell Res.

[22] Kelly KF, Daniel JM (2006). POZ for effect–POZ-ZF transcription factors in cancer and development.. Trends Cell Biol.

[23] Soubry A, van Hengel J, Parthoens E, Colpaert C, Van Marck E (2005). Expression and nuclear location of the transcriptional repressor Kaiso is regulated by the tumor microenvironment.. Cancer Res.

[24] Dai SD, Wang Y, Miao Y, Zhao Y, Zhang Y (2009). Cytoplasmic Kaiso is associated with poor prognosis in non-small cell lung cancer.. BioMed Central Cancer.

[25] Dai SD, Wang Y, Jiang GY, Zhang PX, Dong XJ (2009). Kaiso is expressed in lung cancer: Its expression and localization is affected by p120ctn. Lung Cancer [Epub ahead of print]..

[26] Karsenti E, Vernos I (2001). The mitotic spindle: a self-made machine.. Science.

[27] Walczak CE, Heald R (2008). Mechanisms of mitotic spindle assembly and function.. Int Rev Cytol.

[28] Gunawardane RN, Lizarraga SB, Wiese C, Wilde A, Zheng Y (2000). gamma-Tubulin complexes and their role in microtubule nucleation.. Curr Top Dev Biol.

[29] Zimmerman WC, Sillibourne J, Rosa J, Doxsey SJ (2004). Mitosis-specific anchoring of gamma tubulin complexes by pericentrin controls spindle organization and mitotic entry.. Mol Biol Cell.

[30] Dionne MA, Howard L, Compton DA (1999). NuMA is a component of an insoluble matrix at mitotic spindle poles.. Cell Motil Cytoskeleton.

[31] Kisurina-Evgenieva O, Mack G, Du Q, Macara I, Khodjakov A (2004). Multiple mechanisms regulate NuMA dynamics at spindle poles.. J Cell Sci.

[32] Wittmann T, Wilm M, Karsenti E, Vernos I (2000). TPX2, A novel xenopus MAP involved in spindle pole organization.. J Cell Biol.

[33] Debnath J, Muthuswamy SK, Brugge JS (2003). Morphogenesis and oncogenesis of MCF-10A mammary epithelial acini grown in three-dimensional basement membrane cultures.. Methods.

[34] Daniel JM, Ireton RC, Reynolds AB (2001). Monoclonal antibodies to Kaiso: a novel transcription factor and p120ctn-binding protein.. Hybridoma.

[35] Bostock CJ, Prescott DM, Kirkpatrick JB (1971). An evaluation of the double thymidine block for synchronizing mammalian cells at the G1-S border.. Exp Cell Res.

[36] Tobey RA, Crissman HA (1972). Preparation of large quantities of synchronized mammalian cells in late G1 in the pre-DNA replicative phase of the cell cycle.. Exp Cell Res.

[37] Piel M, Meyer P, Khodjakov A, Rieder CL, Bornens M (2000). The respective contributions of the mother and daughter centrioles to centrosome activity and behavior in vertebrate cells.. J Cell Biol.

[38] Wiznerowicz M, Trono D (2003). Conditional suppression of cellular genes: Lentivirus vector-mediated drug-inducible RNA interference.. J Virol.

[39] Stove V, Van de Walle I, Naessens E, Coene E, Stove C (2005). Human immunodeficiency virus Nef induces rapid internalization of the T cell co-receptor CD8alpha-beta.. J Virol.

[40] Julian M, Tollon Y, Lajoie-Mazenc I, Moisand A, Mazarguil H (1993). gamma-Tubulin participates in the formation of the midbody during cytokinesis in mammalian cells.. J Cell Sci.

[41] Delaval B, Doxsey SJ (2009). Pericentrin in cellular function and disease.. J Cell Biol[Epub ahead of print].

[42] Kelly KF, Otchere AA, Graham M, Daniel JM (2004). Nuclear import of the BTB/POZ transcriptional regulator Kaiso.. J Cell Sci.

[43] Kantidze OL, Kamalyukova IM, Razin SV (2009). Association of the mammalian transcriptional regulator kaiso with centrosomes and the midbody.. Cell Cycle.

[44] Azimzadeh J, Bornens M (2007). Structure and duplication of the centrosome.. J Cell Sci.

[45] Schatten H (2008). The mammalian centrosome and its functional significance.. Histochem Cell Biol.

[46] Sillibourne JE, Delaval B, Redick S, Sinha M, Doxsey SJ (2007). Chromatin remodeling proteins interact with pericentrin to regulate centrosome integrity.. Mol Biol Cell.

[47] Zeng C (2000). NuMA: a nuclear protein involved in mitotic centrosome function.. Microsc Res Tech.

[48] Fant X, Merdes A, Haren L (2004). Cell and molecular biology of spindle poles and NuMA.. Int Rev Cytol.

[49] Ems-McClung SC, Zheng Y, Walczak CE (2004). Importin alpha/beta and Ran-GTP regulate XCTK2 microtubule binding through a bipartite nuclear localization signal.. Mol Biol Cell.

[50] Tokai N, Fujimoto-Nishiyama A, Toyoshima Y, Yonemura S, Tsukita S (1996). Kid, a novel kinesin-like DNA binding protein, is localized to chromosomes and the mitotic spindle.. EMBO J.

[51] Trieselmann N, Armstrong S, Rauw J, Wilde A (2003). Ran modulates spindle assembly by regulating a subset of TPX2 and Kid activities including Aurora A activation.. J Cell Sci.

[52] Yang Z, Gallicano GI, Yu QC, Fuchs E (1997). An unexpected localization of basonuclin in the centrosome, mitochondria, and acrosome of developing spermatids.. J Cell Biol.

[53] Oegema K, Whitfield WG, Alberts B (1995). The cell cycle-dependent localization of the CP190 centrosomal protein is determined by the coordinate action of two separable domains.. J Cell Biol.

[54] Whitfield WG, Chaplin MA, Oegema K, Parry H, Glover DM (1995). The 190 kDa centrosome-associated protein of Drosophila melanogaster contains four zinc finger motifs and binds to specific sites on polytene chromosomes.. J Cell Sci.

[55] Mohan M, Bartkuhn M, Herold M, Philippen A, Heinl N (2007). The Drosophila insulator proteins CTCF and CP190 link enhancer blocking to body patterning.. EMBO J.

[56] Zhang R, Burke LJ, Rasko JE, Lobanenkov V, Renkawitz R (2004). Dynamic association of the mammalian insulator protein CTCF with centrosomes and the midbody.. Exp Cell Res.

[57] Defossez PA, Kelly KF, Filion GJ, Perez-Torrado R, Magdinier F (2005). The human enhancer blocker CTC-binding factor interacts with the transcription factor Kaiso.. J Biol Chem.

[58] Popov AV, Pozniakovsky A, Arnal I, Antony C, Ashford AJ (2001). XMAP215 regulates microtubule dynamics through two distinct domains.. EMBO J.

[59] Gergely F, Draviam VM, Raff JW (2003). The ch-TOG/XMAP215 protein is essential for spindle pole organization in human somatic cells.. Genes Dev.

[60] Young A, Dictenberg JB, Purohit A, Tuft R, Doxsey SJ (2000). Cytoplasmic dynein-mediated assembly of pericentrin and gamma tubulin onto centrosomes.. Mol Biol Cell.

[61] Dammermann A, Merdes A (2002). Assembly of centrosomal proteins and microtubule organization depends on PCM-1.. J Cell Biol.

[62] Franz CM, Ridley AJ (2004). p120 catenin associates with microtubules: inverse relationship between microtubule binding and Rho GTPase regulation.. J Biol Chem.

[63] Chartier NT, Oddou CI, Laine MG, Ducarouge B, Marie CA (2007). Cyclin-dependent kinase 2/cyclin E complex is involved in p120 catenin (p120ctn)-dependent cell growth control: a new role for p120ctn in cancer.. Cancer Res.

[64] Sarrio D, Perez-Mies B, Hardisson D, Moreno-Bueno G, Suarez A (2004). Cytoplasmic localization of p120ctn and E-cadherin loss characterize lobular breast carcinoma from preinvasive to metastatic lesions.. Oncogene.

[65] Bellovin DI, Bates RC, Muzikansky A, Rimm DL, Mercurio AM (2005). Altered localization of p120 catenin during epithelial to mesenchymal transition of colon carcinoma is prognostic for aggressive disease.. Cancer Res.

[66] van Roy FM, McCrea PD (2005). A role for Kaiso-p120ctn complexes in cancer?. Nat Rev Cancer.

[67] Sansom OJ, Maddison K, Clarke AR (2007). Mechanisms of disease: methyl-binding domain proteins as potential therapeutic targets in cancer.. Nat Clin Pract Oncol.

[68] Flory MR, Davis TN (2003). The centrosomal proteins pericentrin and kendrin are encoded by alternatively spliced products of one gene.. Genomics.

[69] Keryer G, Di Fiore B, Celati C, Lechtreck KF, Mogensen M (2003). Part of Ran is associated with AKAP450 at the centrosome: involvement in microtubule-organizing activity.. Mol Biol Cell.

