# Supplementary material for: The Transcriptional Repressor Kaiso Localizes at the Mitotic Spindle and Is a Constituent of the Pericentriolar Material
Source: PLoS One. 2010 Feb 15;5(2):e9203. doi: 10.1371/journal.pone.0009203 (PMC2821401; doi:10.1371/journal.pone.0009203)
Supplement: Figure S1 — Alignment of Kaiso proteins of various species reveals two conserved domains, CD1 and CD2, in addition to the BTB/POZ and zinc finger domains. Multiple sequence alignment of Kaiso protein sequences from mammals (human, chimpanzee, rhesus monkey, horse, mouse, rat) and other vertebrates (chicken, the frog Xenopus laevis, zebrafish). RefSeq sequences were obtained from the Entrez Gene database [1] by searching for “Kaiso OR Zbtb33” and aligned with ClustalX2 using default parameters [2]. The shaded background was created with the BoxShade server. The line below every alignment block denotes the type of conservation: “*” for identical residues, “:” for high similarity and “.” for weak similarity. Annotation of the BTB/POZ domain (cyan background) and zinc fingers (green background; red: C and H residues) was based on, respectively, CD-Search [3] and InterPro [4] analyses. Other conserved regions, CD1, CD2 and NLS, are indicated by yellow background. The novel Spindle-Associated domains 1 and 2 (SA1 and SA2) are highlighted in pink. See also Fig. 1 for domain organization. References [1]: Maglott D, Ostell J, Pruitt KD, Tatusova T (2007) Entrez Gene: gene-centered information at NCBI. Nucleic Acids Res 35: D26-31. [2]: Larkin MA, Blackshields G, Brown NP, Chenna R, McGettigan PA, et al. (2007) Clustal W and Clustal X version 2.0. Bioinformatics 23: 2947–2948. [3]: Marchler-Bauer A, Bryant SH (2004) CD-Search: protein domain annotations on the fly. Nucleic Acids Res 32: W327-331. [4]: Hunter S, Apweiler R, Attwood TK, Bairoch A, Bateman A, et al. (2009) InterPro: the integrative protein signature database. Nucleic Acids Res 37: D211-215. (0.18 MB PDF) [file pone.0009203.s001.pdf]

```
human_Kaiso           1 MESRKLISATDIQYSGSLLNSLNEQRGHGLFCDVTVIVEDRKFRAHKNILSASSTYFHQL
chimpanzee_Kaiso      1 MESRKLISATDIQYSGSLLNSLNEQRGHGLFCDVTVIVEDRKFRAHKNILSASSTYFHQL
rhesus monkey_Kaiso   1 MESRKLISATDIQYSGSLLNSLNEQRGHGLFCDVTVIVEDRKFRAHKNILSASSTYFHQL
horse_Kaiso           1 MESRKLISATDIQYSGSLLNSLNEQRGHGLFCDVTVIVEDRKFRAHRNILSASSTYFHQL
mouse_Kaiso           1 MESRKLISATDIQYSASLLNSLNEQRGHGLFCDVTVIVEDRKFRAHRNILSASSTYFHQL
rat_Kaiso             1 MESRKLISATDIQYSASLLNSLNEQRGHGLFCDVTVIVEDRKFRAHRNILSASSTYFHQL
chicken_Kaiso         1 MEGEKLISATDTQYSSSLLQSLNEQRGHGLFCDVTVIVEDRKFRAHRNILSASSTYFHQL
frog_Kaiso            1 METKKLITATDTQYSGILLNALNDQRIQGLYCDVTVIVEDRKFRAHRNILSACSTYFHQL
zebrafish_Kaiso       1 MSKLKLISVVDTQYPGSLLKNINDERHNGLFCDVTIVVQDRKFRAHKCVLSASSTYFRQL
consensus             1 *...***...*.**...**...*..*..**.****..*.*******...***.****.**
                                   <            BTB/POZ domain                     .

human_Kaiso          61 FSVAGQVVELSFIRAEIFAEILNYIYSSKIVRVRSDLLDELIKSGQLLGVKFIAELGVPL
chimpanzee_Kaiso     61 FSVAGQVVELSFIRAEIFAEILNYIYSSKIVRVRSDLLDELIKSGQLLGVKFIAELGVPL
rhesus monkey_Kaiso  61 FSVAGQVVELSFIRAEIFAEILNYIYSSKIVRVRSDLLDELIKSGQLLGVKFIAELGVPL
horse_Kaiso          61 FLVAGQVVELSFIRAEIFAEILNYIYSSKIVRVRSDLLDELIKSGQLLGVKFIAELGVPL
mouse_Kaiso          61 FSVAGQVVELSFIRAEIFAEILNYIYSSKVVRVRADLLDELIKSGQLLGVKFIAELGVPL
rat_Kaiso            61 FSVAGQVVELSFIRAEIFAEILNYIYSSKIVRVRADLLDELIKSGQLLGVKFIAELGVPL
chicken_Kaiso        61 LSVAGQVVELSFVRADIFAEILNYMYSSKIISVRADLIDELIKSGQQLGVKFLADLSVAP
frog_Kaiso           61 FSVAGQVVELNFVKADIFAEILNYIYSSKIVRVRCDMLEELIKSGKLLGVPFIAELGIPL
zebrafish_Kaiso      61 FSVAGKVVELNFIKADIFDMVLNYIYTSQMFRVRSDRLEDLINAGQILGVTFIANLAIPL
consensus            61 ..***.****.*..*.**...***.*.*....**.*....**..*..***.*.*.*....
                        .                       BTB/POZ domain                  >

human_Kaiso         121 SQVKSISGTAQDGNTEPLPPDSGDKNLVIQKSKDE------AQDNGAT-IMPIITESFSL
chimpanzee_Kaiso    121 SQVKSISGTAQDGNTEPLPPDSGDKNLVIQKSKDE------AQDNGAT-IMPIITESFSL
rhesus monkey_Kaiso 121 SQVKSISGTAQDGNTEPLPPDSGDKNLVIQKSKDE------AQDNGAT-IMPIITESFSL
horse_Kaiso         121 SQVKSISGTAQDGNTDTLPPGSSDKNLEIQKSKDE------AQDNGAT-IMPIITESFSL
mouse_Kaiso         121 SQVKSISGTEQDGTAETLPSSSSDKSLDMEKSKDE------AQDNGAT-VMPIITESFSL
rat_Kaiso           121 SQVKSISGTVQDGTAETLPSSSNDKSPEIEKSKDE------AQDNGAP-VMPIITESFSL
chicken_Kaiso       121 SDGRSVPGEVKDGASGTSGSAPTQKDAETQVPAIR------QEGREVTGWMPVITESFSL
frog_Kaiso          121 SQVKSISGAGGKDGGTDAPSNPDHKAPEPQKSSDSPLPCTVKIKADVKTEMPVITESFSL
zebrafish_Kaiso     121 AQVKGLPGLSKDGDNNIVSS--------PEKPEDG----------GVN--MPIITETFSL
consensus           121 .......*..... .. ... . .. .........      ....... .**.***.***
                                                                          <  CD1    .

human_Kaiso         174 SAEDYEMKKIIVTD----SDDDDDD-VIFCSEILPTKETLPSNNTVAQVQSNPGPVAISD
chimpanzee_Kaiso    174 SAEDYEMKKIIVTD----SDDDDDD-VIFCSEILPTKETLPSNNTVTQVQSNPGPVAISD
rhesus monkey_Kaiso 174 SAEDYEMKKIIVTD----SDDDDDD-VIFCSEILPTKETLPSNNTVAQVQSNPGPVAISD
horse_Kaiso         174 SAEDYETKKIIVTD----SDDDDDD-VIFCSEILPAKETLPSTNAVAQVQPNPGAVAISD
mouse_Kaiso         174 SAEDNEMKKIIVTD----SDDDDDDDVIFCSEILPAKEDLPSNNTATQVQPNPASVAISE
rat_Kaiso           174 SAEDYEMKKIIVTD----SDDDDDDDVIFCSEILPAKEDLPSNNTATQVQPNPASVAISE
chicken_Kaiso       175 HGIAYETPKITVTD----SDD-DDD-VIFCSEIVPPKECTKDSSAAIQNQPCPNASGASD
frog_Kaiso          181 SSDDYKDKKASGSQDHNSEKEDDDDDVIFCSEIVSSKQAPAERKEAAQTQIPPDNEQVPE
zebrafish_Kaiso     161 SAEEFNMTGANTEK-----EDSDSDDVLFVSKTEAP---QAQKTKISDVHKNIEGAPETK
consensus           181 ..............    ....*.* *.*.*.... .. ....... ... ... .....
                        .        CD1                  >             <       SA1     .

human_Kaiso         229 VAPSASNNSPPLTNITPTQKLPTPVNQATLSQTQGS-EKLLVSSAPTHLTPNIILLNQTP
chimpanzee_Kaiso    229 VAPSASNNSPPLTNITPTQKLPTPVNQATLSQTQGS-EKLLVSSAPTHLTPNIILLNQTP
rhesus monkey_Kaiso 229 VAPSASNNSPPLTNITPTQKLPTPVNQATLSQTQGS-EKLLVSSAPTHLTPNIILLNQTP
horse_Kaiso         229 VAPCVSNNSPPLTNITPTQKLPTSANQATLSQTQGS-EKLLVSSAPTHLTPNIILLNQAS
mouse_Kaiso         230 VTPCASNNSPPVTNITPTQ-LPTPVNQATLSQTQGS-EELLVSSASTHLTPNIILLNQAP
rat_Kaiso           230 VTPCTSSNSPPVTNITPPQ-LPTPVNQSTLSQAQGS-EELLVSSASTHLTPNIILLNQAP
chicken_Kaiso       229 QKSCGSGGSPLLTSTTSTQKLTSSANQLNPNQTLSS-AESLVPVAPQHSTTELILLNRPP
frog_Kaiso          241 VKKVTPSSQVQLTQNSLPTNQQSSKNTSSTTQKFTPPVNANISKNPTPAANGFLSPTAQK
zebrafish_Kaiso     213 KQKVTNEEG-VAPKSSIPQEKKSSERNASLQNNLDP--DVVILSSPEKSSSILTSPVGTG
consensus           241 . . ................................ . ................... .
                        .          SA1                     >

human_Kaiso         288 LSTPPNVSSSLPNHMPSSINLLVQNQQTPNSAILTGNKANEEEEEEIIDDDDDTISSSPD
chimpanzee_Kaiso    288 LSTPPNVSSSLPNHMPSSINLLVQNQQTPNSAILTGNKANEEEEEEIIDDDDDTISSSPD
rhesus monkey_Kaiso 288 LSTPPNVSSSLPNHIPSSINLLVQNQQTPNSAILTGNKANEEEEEEIIDDDDDTISSSPD
horse_Kaiso         288 HTTPPNVSSSLPNHMSPSINLLVQNQPTPNSAVLTGNKANEEEEEEIIDDDDDTISSSPD
mouse_Kaiso         288 LTAPPSASSSLPNHMSSSVNVLVQNQQTPNSAVLTGNKA--EEEEEIIDDDDDIISSSPD
rat_Kaiso           288 LTAPPSVSSSLPNHMSSSVNVLVQNQQAPNSAV-TGNKA--EEEEEILDDDDDIISSSPD
chicken_Kaiso       288 VNAPP--SSSHQTHVPPTIDLVEESQKPSDKGSSTERGTAAVDVEEVVEDSDDVISSSSP
frog_Kaiso          301 QGTPNAVQNQHSQNITSGNALPQQKPVVNFSSIKPQQISAIKPKTEVIIHGNGLSPPSS-
zebrafish_Kaiso     270 -------SNSNPKPPACPDSVPSD----------PQNVSLPSDNIEVLG-----------
consensus           301 ..... ......... .......................  ....*....... ......
                                                                               <   .

human_Kaiso         348 SAVSNTSLVPQADTSQNTSFDGSLIQKMQIPTLLQEPLSNS----LKISDIITRNTN--D
chimpanzee_Kaiso    348 SAVSNTSLVPQADTSQNTSFDGSLIQKMQIPTLLQEPLSNS----LKISDIITRNTN--D
rhesus monkey_Kaiso 348 SAVSNTSLVPQADTSQNTSFDGSLIQKMQIPTLLQEPLSNS----LKISDIITRNTN--D
horse_Kaiso         348 SAVSNTSLVPQADIPQNTTFDGSLIQKMQIPTLLQEPLSNS----LKISDIITRNTN--D
mouse_Kaiso         346 SAVSNTSLVPQADNSKSTTLDGSLTQKMQIPVLPQEPPSNS----LKISDVITRNTN--D
rat_Kaiso           345 SAVSNTSLVPQADASKSTTFDGSLTQKMQAPILPQEPPSNS----LKISDVITRNTN--D
chicken_Kaiso       346 SSVSNSSSV-QPSVPKAAPSEGSGVQKKQVVTFPQEQSSKPGEFKIKISDVLSGNNKEFG
frog_Kaiso          360 ---SVIPLGQQPVTPKHISFDG--VQKKQVVTFTQGSPSKPGEFKIKIADVVSGSSLDSF
zebrafish_Kaiso     302 ------------------------VQKK--KVLLEHSPDHPG--KITLSDVRSSCSS-SN
consensus           361 ............. .  ........**.......... ...    ....*.......  .
                        .                        SA2                                .
```

```
human_Kaiso           402 PGVGSKHLMEGQKIITLDTATEIEGLSTGCKVYANIGEDTYDIVIPVKDDPDEG-----E
chimpanzee_Kaiso      402 PGVGSKHLMEGQKIITLDTATEIEGLSTGCKVYANIGEDTYDIVIPVKDDPDEG-----E
rhesus monkey_Kaiso   402 PGLGSKHLMEGQKIITLDTATEIEGLSTGCKVYANIGEDTYDIVIPVKDDPDEG-----E
horse_Kaiso           402 PGLGSKHLMEGQKIITLDTATEIEGLSTGCKVYANIGEDTYDIVIPVKDDPDEG-----E
mouse_Kaiso           400 PGLRSKHVMEGQKIITLDTATEIEGLSTGCKVYANIGEDTYDIVIPVKDDPDGG-----E
rat_Kaiso             399 PGLRSKHVMEGQKIITLDTATEIEGLSTGCKVYANIGEDTYDIVIPVKDDPDGG-----E
chicken_Kaiso         405 PGIASKHAGEGQKIITLDTATEIEGLSTGCKVYANIGEDTYDIVIPIKDDPEEG-----E
frog_Kaiso            415 KDSEPRRIIDGKKIITLDTASEIEGLSTGCKVYANIGEDTYDIVIPIKEDPEEG-----E
zebrafish_Kaiso       333 GPTNVIPALSVKKTITLDKASEIDSLSPGCKVYANIGENTYDIVP-VKDDPGEGAAPGCK
consensus             421 ............*.****.*.**..**.**********.*****...*.**..*     .
                          .                    CD2                                    .

human_Kaiso           457 ARLENEIPKTS-GSEMANKRMKVKHDDHYELIVDGRVYYICIVCKRSYVCLTSLRRHFNI
chimpanzee_Kaiso      457 ARLENEIPKTS-GSEMANKRMKVKHDDHYELIVDGRVYYICIVCKRSYVCLTSLRRHFNI
rhesus monkey_Kaiso   457 ARLENEIPKTS-GSEMANKRMKVKHDDHYELIVDGRVYYICIVCKRSYVCLTSLRRHFNI
horse_Kaiso           457 ARLENETPKTS-SSETANKRMKVKHDDHYELIVDGRVYYICIVCKRSYVCLTSLRRHFNI
mouse_Kaiso           455 AKLDNELPKTS-GSEPPNKRMKVKHDDHYELIVDGRVYYICIVCKRSYVCLTSLRRHFNI
rat_Kaiso             454 AKLENELPKTS-GSEPSNKRMKVKHDDHYELIVDGRVYYICIVCKRSYVCLTSLRRHFNI
chicken_Kaiso         460 AK-PNEAPRTSDGDSTSRKRMKVKHDDHYELIVDGRVYYICIVCKRSYVCLTSLRRHFNV
frog_Kaiso            470 AKLDLDG-------LPNRKRMKLKHDDHYELIVDGRVYYICIVCKRSYVCLTSLRRHFNV
zebrafish_Kaiso       392 SQIQPDSPNTASLSPKSKKKFKPDQEDHYEIIMDGKTFFVCIVCKRPYVCLTSLRRHFNT
consensus             481 ........... ...  .*..*....****.*.**.....******.************.
                          .  SA2               NLS               ><                   .

human_Kaiso           516 HSWEKKYPCRYCEKVFPLAEYRTKHEIHHTGERRYQCLACGKSFINYQFMSSHIKSVHSQ
chimpanzee_Kaiso      516 HSWEKKYPCRYCEKVFPLAEYRTKHEIHHTGERRYQCLACGKSFINYQFMSSHIKSVHSQ
rhesus monkey_Kaiso   516 HSWEKKYPCRYCEKVFPLAEYRTKHEIHHTGERRYQCLACGKSFINYQFMSSHIKSVHSQ
horse_Kaiso           516 HSWEKKYPCRYCEKVFPLAEYRTKHEIHHTGERRYQCLACGKSFINYQFMSSHIKSVHSQ
mouse_Kaiso           514 HSWEKKYQCRYCDKVFPLAEYRTKHEIHHTGERRYQCLTCGKSFINYQFMSSHIKSVHSQ
rat_Kaiso             513 HSWEKKYQCRYCDKVFPLAEYRTKHEIHHTGERRYQCLACGKSFINYQFMSSHIKSVHSQ
chicken_Kaiso         519 HSWEKKYPCRYCDKVFPLAEYRTKHEIHHTGERRYQCLTCGKSFINYQIMASHIRSVHSQ
frog_Kaiso            523 HSWEKKYPCRYCERVFPLAEYRTKHEIHHTGERRYQCLTCGSSFINYQVMASHIRSVHSL
zebrafish_Kaiso       452 HSWERKYPCHYCNKVFALAEYRTKHEISHTGERRYQCLLCNEMFLNYQILSTHCKQAHNQ
consensus             541 ****.**.*.**..**.**********.**********.*...*.***....*....*..
                          .              3 x C2H2 Zinc finger domains               >

human_Kaiso           576 DPSG-------DSKLYRLHPCRSLQIRQYAYLSDRSS-------------------TIPA
chimpanzee_Kaiso      576 DPSG-------DSKLYRLHPCRSLQIRQYAYLSDRSS-------------------TIPA
rhesus monkey_Kaiso   576 DPSG-------DSKLYRLHPCRSLQIRQYAYLSDRSS-------------------TMPA
horse_Kaiso           576 DPSG-------DSKLYRLHPCRSLQIRQYAYLSDRSG-------------------TMPV
mouse_Kaiso           574 DPSG-------DSKLYRLHPCKSLQIRQYAYLSNKSS-------------------AMPV
rat_Kaiso             573 DPSG-------DSKLYRLHPCKSLQIRQYAYLSNKPS-------------------AMPV
chicken_Kaiso         579 DPSG-------DNKLYRLHPCKSLQIRQYAYISDRSS-------------------SLPV
frog_Kaiso            583 DPSG-------DSKLYRLNPCKTLQIRQYAYVNNSTNGTVINDGAINVPVITDGGINVPV
zebrafish_Kaiso       512 DPSGRKEKDDSANNLYRLLPCKTVQFKPYSFVSDESG-------------------GSPV
consensus             601 ****       ...****.**...*...*........                   ..*.

human_Kaiso           610 MKDDGIGYKVDTG---KEPPVGTTTST-QNKPMTWEDIFIQQEND--------SIFKQNV
chimpanzee_Kaiso      610 MKDDGIGYKVDTG---KEPPVGTTTST-QNKPMTWEDIFIQQEND--------SIFKQNV
rhesus monkey_Kaiso   610 MKGDGIGYKVDTG---KEPPVGTTTST-QNKPMTWEDIFIQQEND--------SIFKQNV
horse_Kaiso           610 MKDDGIGYKVDAG---KEPPVGTTSTP-QNKPMTWEDIFIQQEND--------SIFKQNV
mouse_Kaiso           608 MKDDAVGYKVDAG---KEPPVGTTSTPPQNKSTFWEDIFIQQEND--------SIFKQNV
rat_Kaiso             607 MKDDAVGYKVDAG---KEPPVGTTSAPPQNKSTFWEDIFIQQEND--------SIFKQNV
chicken_Kaiso         613 INENRIVYRVDAA---KDGTEGTTSNS-PGKQMSWDDIFVPQGND--------AIFKQNQ
frog_Kaiso            636 INDGGIVYDIDPDEPQQPASEGNHANS-ATKPVNWDNIFIQQSNQ--------NMFKLNT
zebrafish_Kaiso       553 IGEDGTIQYVNSE---KEHCTNQPLPSNQSKALNWDDIFVESEGQNARPAPPPQPVDQIN
consensus             661 ........... .   ........... ..*...*..**......        .......

human_Kaiso           658 TDGSTEFEFIIPESY
chimpanzee_Kaiso      658 TDGSTEFEFIIPESY
rhesus monkey_Kaiso   658 TDGSTEFEFIIPESY
horse_Kaiso           658 TDGSTEFEFIIPESY
mouse_Kaiso           657 TDGSTEFEFIIPESY
rat_Kaiso             656 TDGSTEFEFIIPESY
chicken_Kaiso         661 SEGSTEFEFVIPESY
frog_Kaiso            687 SEGGTEFEFVIPESY
zebrafish_Kaiso       610 APARNEFEFVIPETY
consensus             721 .....****.***.*
```

Suppl. Fig. S1: Alignment of Kaiso proteins of various species reveals two conserved domains, CD1 and CD2, in addition to the BTB/POZ and zinc finger domains. Multiple sequence alignment of Kaiso protein sequences from mammals (human, chimpanzee, rhesus monkey, horse, mouse, rat) and other vertebrates (chicken, the frog *Xenopus laevis*, zebrafish). RefSeq sequences were obtained from the Entrez Gene database [1] by searching for "Kaiso OR Zbtb33" and aligned with ClustalX2 using default parameters [2]. The shaded background was created with the BoxShade server. The line below every alignment block denotes the type of conservation: "*" for identical residues, ":" for high similarity and "." for weak similarity. Annotation of the BTB/POZ domain (cyan background) and zinc fingers (green background; red: C and H residues) was based on, respectively, CD-Search [3] and InterPro [4] analyses. Other conserved regions, CD1, CD2 and NLS, are indicated by yellow background. The novel Spindle-Associated domains 1 and 2 (SA1 and SA2) are highlighted in pink. See also Fig. 1 for domain organization.

References

1. Maglott D, Ostell J, Pruitt KD, Tatusova T (2007) Entrez Gene: gene-centered information at NCBI. Nucleic Acids Res 35: D26-31.
2. Larkin MA, Blackshields G, Brown NP, Chenna R, McGettigan PA, et al. (2007) Clustal W and Clustal X version 2.0. Bioinformatics 23: 2947-2948.
3. Marchler-Bauer A, Bryant SH (2004) CD-Search: protein domain annotations on the fly. Nucleic Acids Res 32: W327-331.
4. Hunter S, Apweiler R, Attwood TK, Bairoch A, Bateman A, et al. (2009) InterPro: the integrative protein signature database. Nucleic Acids Res 37: D211-215.
