# Supplementary material for: The Transcriptional Repressor Kaiso Localizes at the Mitotic Spindle and Is a Constituent of the Pericentriolar Material
Source: PLoS One. 2010 Feb 15;5(2):e9203. doi: 10.1371/journal.pone.0009203 (PMC2821401; doi:10.1371/journal.pone.0009203)
Supplement: Figure S3 — The GFP-tagged Kaiso fragment K4z localizes at spindle material during mitosis. During metaphase (upper row) and anaphase (lower row) of transfected HEK293 cells, the Kaiso fragment K4z concentrates at the centrosomes (arrowheads) and the minus ends of the spindle microtubules (arrows). DNA was stained with DAPI and cells were imaged with a Leica DM IRE2 microscope (63× objective lens). (1.59 MB PDF) [file pone.0009203.s003.pdf]

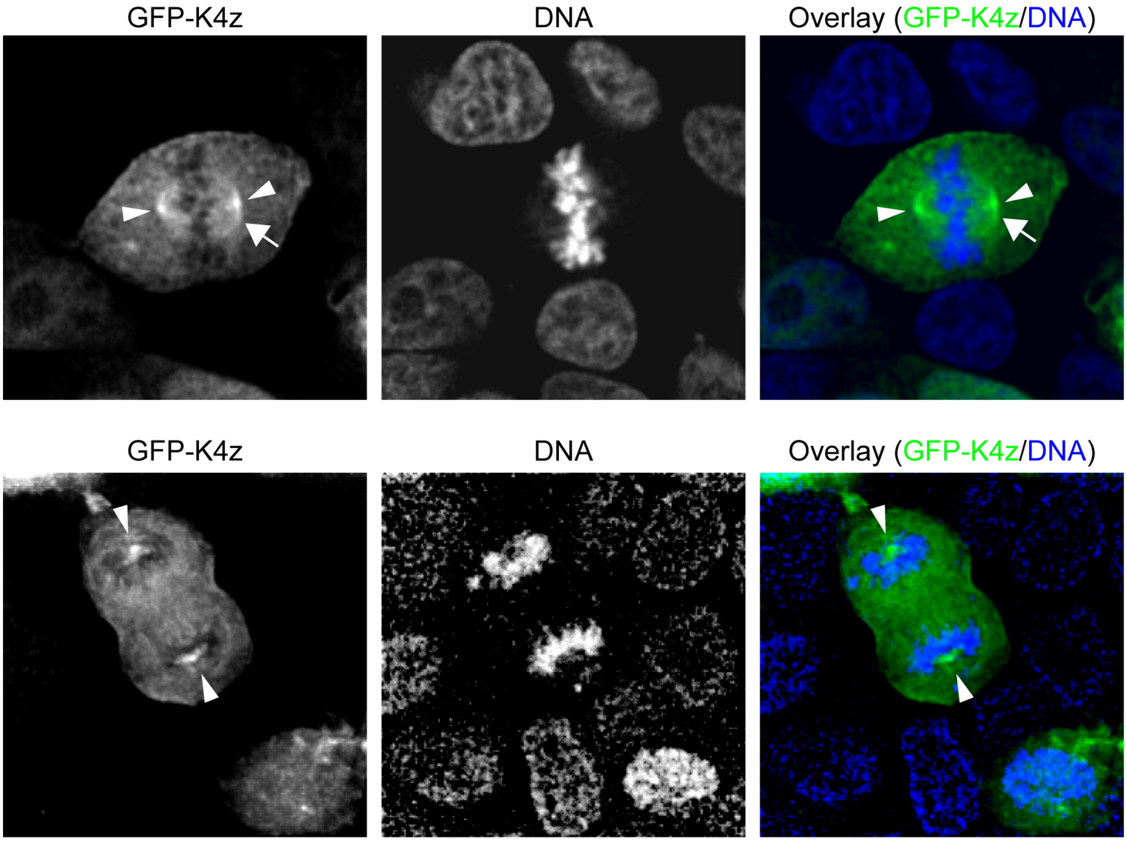

Suppl. Fig. S3: The GFP-tagged Kaiso fragment K4z localizes at spindle material during mitosis. During metaphase (upper row) and anaphase (lower row) of transfected HEK293 cells, the Kaiso fragment K4z concentrates at the centrosomes (arrowheads) and the minus ends of the spindle microtubules (arrows). DNA was stained with DAPI and cells were imaged with a Leica DM IRE2 microscope (63X objective lens).
