# Supplementary material for: The Transcriptional Repressor Kaiso Localizes at the Mitotic Spindle and Is a Constituent of the Pericentriolar Material
Source: PLoS One. 2010 Feb 15;5(2):e9203. doi: 10.1371/journal.pone.0009203 (PMC2821401; doi:10.1371/journal.pone.0009203)
Supplement: Figure S4 — The GFP-tagged Kaiso fragments K4a and K4b localize at the spindle material during mitosis. In transfected HEK293 cells the K4a (upper row) and K4b (lower row) Kaiso fragments both localize at the spindle microtubules (arrows) and at the centrosomes (arrowheads). DNA was stained with DAPI and cells were imaged with a Zeiss Axiophot microscope (100× objective lens). (0.70 MB PDF) [file pone.0009203.s004.pdf]

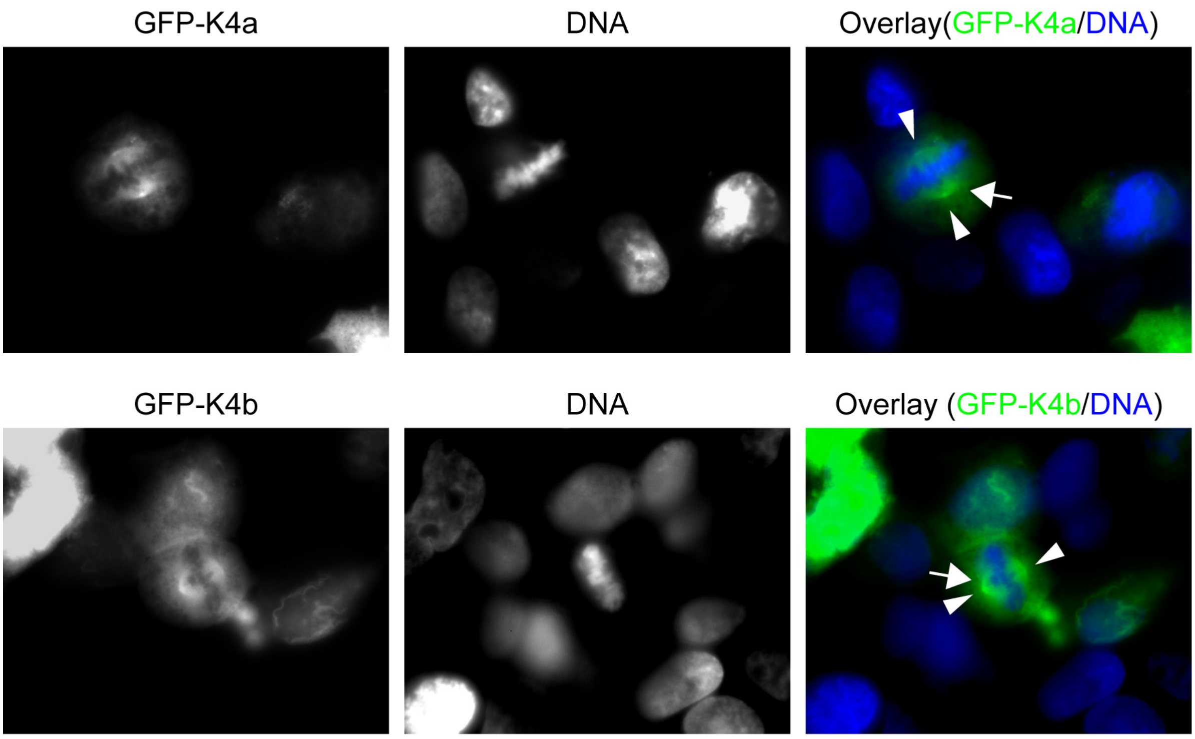

Suppl. Fig. S4: The GFP-tagged Kaiso fragments K4a and K4b localize at the spindle material during mitosis. In transfected HEK293 cells the K4a (upper row) and K4b (lower row) Kaiso fragments both localize at the spindle microtubules (arrows) and at the centrosomes (arrowheads). DNA was stained with DAPI and cells were imaged with a Zeiss Axiophot microscope (100X objective lens).
